# Experimental analysis of bone marrow adipose tissue and bone marrow adipocytes: An update from the bone marrow adiposity society (BMAS)

**DOI:** 10.1016/j.bonr.2025.101861

**Published:** 2025-07-28

**Authors:** Michaela Tencerova, Biagio Palmisano, Stéphanie Lucas, Camille Attané, Kaisa K. Ivaska, Léa Loisay, Yoshiko M. Ikushima, Drenka Trivanovic, Alessandro Corsi, Adriana Roque, Hongshuai Li, Friederike Behler-Janbeck, Jeroen Geurts, Mara Riminucci, Izabela Podgorski, William P. Cawthorn, Bram C.J. van der Eerden, André J. van Wijnen

**Affiliations:** a Laboratory of Molecular Physiology of Bone, Institute of Physiology of the Czech Academy of Sciences, Prague, Czechia; b Department of Molecular Medicine, Sapienza University, Rome, Italy; c Marrow Adiposity and Bone Lab, MABLab-ULR4490, Univ. Littoral Côte d'Opale F-62200 Boulogne-sur-Mer, Univ. Lille F-59000 Lille, CHU Lille, F-59000 Lille, France; d Institut de Pharmacologie et de Biologie Structurale (IPBS), Université de Toulouse, CNRS UMR 5089, 205 Route de Narbonne, 31077 Toulouse, France; e Institute of Biomedicine, University of Turku, Turku, Finland; f Rheumatology, Department of Musculoskeletal Medicine, University Hospital Lausanne and University of Lausanne (CHUV-UNIL), Lausanne, Switzerland; g Department of Medical Science and Innovation, SiRIUS Institute of Medical Research, Tohoku University, 1-1 Seiryo-machi, Aoba-ku, Sendai City, Miyagi, 980-8574, Japan; h Group for Hematology and Stem Cells, Institute for Medical Research, National Institute of Republic of Serbia, University of Belgrade, 11000 Belgrade, Serbia; i Clinical Hematology Department, Centro Hospitalar e Universitário de Coimbra (CHUC), Coimbra, Portugal; j Department of Orthopedics and Rehabilitation, University of Iowa, Iowa City, IA, United States; k Department of Biochemistry and Molecular Cell Biology, University Medical Center Hamburg-Eppendorf, Hamburg, Germany; l Department of Pharmacology, Wayne State University School of Medicine and Karmanos Cancer Institute, Detroit, MI, United States; m University/BHF Centre for Cardiovascular Science, University of Edinburgh, The Queen's Medical Research Institute, Edinburgh BioQuarter, 47 Little France Crescent, Edinburgh, UK; n Laboratory for Calcium and Bone Metabolism and Erasmus MC Bone Centre, Department of Internal Medicine, Erasmus MC, Erasmus University Medical Center, Rotterdam, the Netherlands; o Department of Biochemistry, Lerner College of Medicine, 89 Beaumont Avenue, University of Vermont, Burlington, VT, USA

## Abstract

Bone marrow adipose tissue (BMAT) is physiologically linked to bone and energy metabolism, endocrine regulation, hematopoiesis and cancer-related processes. A key challenge in the field is that methods for isolating BMAT or bone marrow adipocytes (BMAds) are variable because there are no widely adopted standardized protocols. To generate awareness of this challenge and to establish uniformity in experimental approaches requiring isolation, storage and characterization of BMAT and BMAds, the Biobanking Working Group of the international Bone Marrow Adiposity Society (BMAS) has previously recommended experimental standards. This paper provides an update on this effort and presents current state-of-the-art methods and technical considerations for isolation and characterization of BMAT and BMAds, including currently available high-throughput omics approaches. This review provides a reference point based on the consensus view of BMAS investigators to support studies on biomedical, biological, biochemical and biophysical questions associated with bone marrow adiposity.

## Highlights

• Bone marrow adipose tissue (BMAT) is critical for energy metabolism
• BMAT supports bone homeostasis, endocrine regulation and hematopoiesis
• BMAT and bone marrow adipocytes are dynamic and diverse across bone sites and species
• Standardization, rigor and transparency is required to harmonize studies on BMAS
• This review provides a consensus view for standards and technical considerations

## Abbreviations

**Fig. 1:**
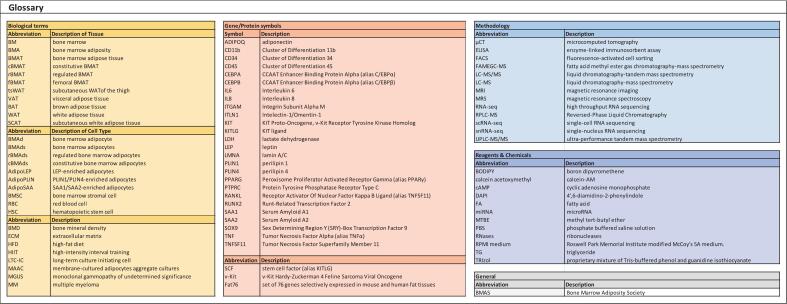
Glossary of abbreviations for biological terms (e.g., tissues and cell types), gene/protein symbols, methodologies, reagents and chemical compounds discussed in the main text.

(see Glossary in Fig. 1)

## 1 Introduction

This paper provides a brief synopsis of the fundamentals of bone marrow adiposity (BMA), as well as general methods for the isolation and analyses of bone marrow adipose tissue (BMAT) and bone marrow adipocytes (BMAds). A glossary is provided to guide the reader with general abbreviations and acronyms for biological terms recommended by BMAS (Bravenboer et al., 2019) (Fig. 1). The narrative concludes with guidelines for recently developed methodologies and omics approaches, as well as future perspectives and key unresolved questions in the BMA field that can be experimentally addressed with advancements in technologies that offer high-throughput analyses at higher molecular and/or microscopic resolution.

### 1.1 Cellular heterogeneity of bone marrow

Mammalian bone marrow (BM) is one of the largest organs within the body. It is located in the central medullary cavity and in the intertrabecular spaces of long bones including the tibia, femur or humerus, and axial bones such as vertebrae and pelvis. It is a heterogeneous tissue and contains a diverse range of hematopoietic cells (e.g., erythrocytes, lymphocytes, macrophages, megakaryocytes, natural killer cells) and non-hematopoietic cells (e.g., stromal cells, reticular cells, skeletal stem cells, BMAds), as well as cells that directly maintain bone homeostasis, including osteoclasts and osteoblasts that line the cortical and trabecular bone surfaces. The remaining BM components include nerve fibers and vascular cells that supply nutrients to the BM (Vogler 3rd and Murphy, 1988; Karampinos et al., 2018; Compston, 2002). The relative amount of BMAT in the marrow space differs among species: the volume of BMAT relative to the bone volume is proportionally greater in larger versus smaller animals (i.e., human > rabbits > rats > mice) (Scheller et al., 2015).

### 1.2 Age-related transitions from red to yellow bone marrow

**Fig. 2:**
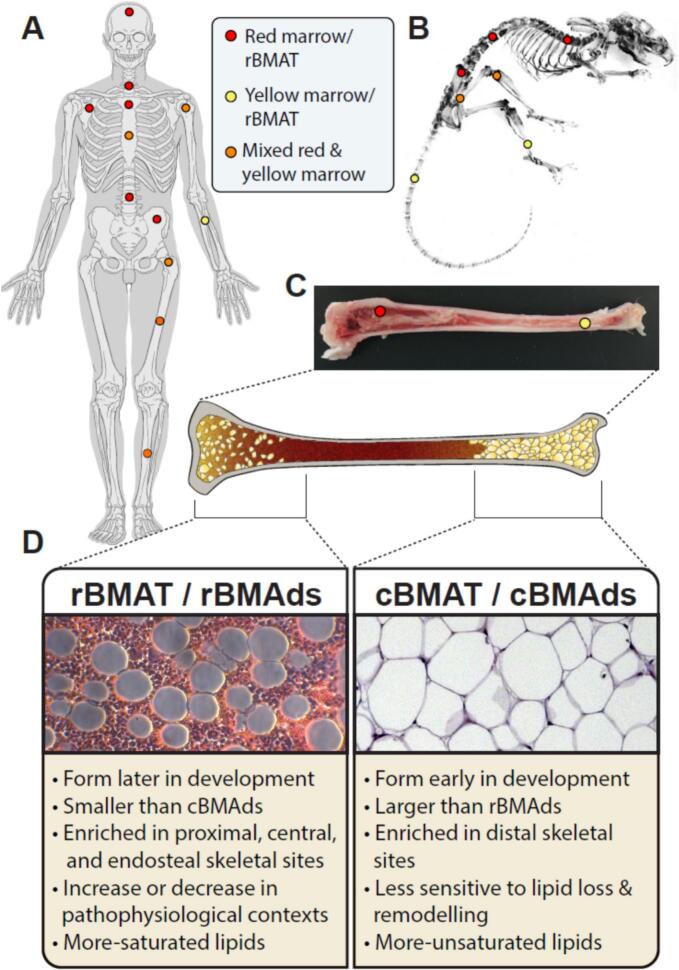
**Schematic overview of red and yellow marrow locations within the mammalian skeleton.** Red or yellow bone marrow deposits, or a mix of the two are found in different bones throughout the mammalian skeleton as exemplified for human (A) or mouse (B). Within long bones of rabbits, red and yellow marrow are typically observed at opposite ends (C) and have different biological properties (D). The image is inspired by elements presented in a previous review paper showing examples of rBMAT and CBMAT in rabbits (Cawthorn et al., 2016). The proximal epiphysis and mid diaphysis both contain a mix of yellow and red BM (Attane et al., 2021).

At birth, tissue within the BM cavity of human bones is mainly composed of active hematopoietic cells and few BMAds. This tissue is referred to as “red marrow” (i.e., red BM) due to the red color conferred by the presence of hemoglobin in erythrocytes. Red BM consists of approximately 40–60 % lipids, 30–40 % water and 10–20 % of protein. The function of red BM is the delivery of blood cells throughout the body. It has a rich vasculature composed of a huge network of sinusoids. In healthy adults, red BM is found in the cavities of skull, scapulae, vertebrae, ribs, and pelvic bones (Fig. 2A). The sternum and ends of the long bones also contain red marrow, but this type of marrow is more accurately described as ‘mixed’ red-yellow BM, because it has a higher lipid content than red BM at other skeletal sites (Fig. 2A) (Suchacki et al., 2020). During growth and aging, hematopoietic cells are gradually replaced by adipocytes within the BM, resulting in a yellow appearance due to the accumulation of carotenoid derivatives that are dissolved in fat droplets of adipocytes (Karampinos et al., 2018; Vande Berg et al., 1998; Lecka-Czernik et al., 2017; Li et al., 2018). This conversion from red to yellow marrow displays a centripetal pattern, starting from the terminal phalanges to the appendicular skeleton and finally up to the axial skeleton (including spine, sternum, ribs, pelvis as well as skull) and within the long bones from diaphysis to metaphysis (Moore and Dawson, 1990).

When healthy humans reach peak bone mass (typically by the age of 25), the volume of BMAT makes up approximately 70 % of the marrow volume, which represents more than 8 % of total fat mass. Compared to the chemical composition of red marrow, yellow BM has a lower water and protein content (∼15 % water and 5 % protein) (Karampinos et al., 2018; Justesen et al., 2001). Later in life, conversion from red to yellow marrow continues at a slower rate (Li et al., 2018). In general, the percentage BMAT positively correlates with age (Justesen et al., 2001), as was recently further evidenced by analysis of a large cohort of individuals (*n* > 46,000) in the UK Biobank (Xu et al., 2025). This temporal conversion of red to yellow BM, which has been clinically observed in humans, is also thought to apply to other mammals and lower vertebrates. Hence, experimental protocols for isolating tissues, cells and molecules from BM should consider the dynamic temporal changes in its heterogeneous composition.

### 1.3 Regulated and constitutive bone marrow adipose tissue and adipocytes

Similar to white adipose tissue (WAT) and brown adipose tissue (BAT), BMAT is highly heterogeneous in terms of location, function, and energy metabolism (Scheller et al., 2015; Tavassoli et al., 1977). Recent studies introduced the concepts of regulated BMAT (rBMAT) and constitutive BMAT (cBMAT), differentiated by the time of formation during development and their responses to pathophysiological cues, such as aging, insulin, and cold (Fig. 2D) (Bravenboer et al., 2019; Vogler 3rd and Murphy, 1988; Scheller et al., 2015; Craft et al., 2018). cBMAT, forming early during mammalian development (present in rodents and humans), typically appears as densely packed groups of large adipocytes with minimal hematopoietic cell infiltration, showing resistance to many pathological cues. Evidence obtained with human specimens in the UK Biobank revealed that cBMAT increases with aging (e.g. in femoral diaphysis and spine) (Xu et al., 2025). In contrast, rBMAT develops gradually and later during post-natal development and shows a more-pronounced accumulation with aging. rBMAT is characterized by single cells interspersed within the hematopoietic BM and responds dynamically to pathophysiological stimuli reflecting its plasticity in responding to changes in metabolic conditions (Fig. 2D) (Scheller et al., 2015; Craft et al., 2018). Although rBMAT and cBMAT were defined by function, these tissue types have relatively specific defined anatomical locations that vary across species. In rodents, rBMAT is generally enriched in the proximal and central parts of the skeleton. For example, rBMAT is found above the tibia/fibula junction and extends into the femur, pelvis, sternum, ribs, and thoracic/lumbar vertebrae (Fig. 2B). On the other hand, cBMAT is more prevalent in distal skeletal regions like the hands, feet, distal tibiae, and tail vertebrae (Fig. 2B). A core of cBMA-like yellow marrow runs through the center of the femur and tibia in rabbits, and this core is surrounded by a ring of red marrow with interspersed adipocytes (Fig. 2C) (Cawthorn et al., 2016). In humans, a similar type of macroscopic organization has been observed in proximal femoral metaphyses and diaphyses (Attane et al., 2021).The physiological distinctions between red and yellow BM, which differ in the relative presence of fat versus hematopoietic tissue in larger mammals, are reflected by their anatomical location. One notion in the field is that yellow BM may fill the mid-diaphysis of the femur during later stages of human development into adulthood. Other data indicate that the proximal epiphysis and mid diaphysis contain a mix of yellow and red BM (Attane et al., 2021).

Analogous to the distinction between regulated and constitutive BMAT, there are two principal subtypes of adipocytes in BMAT, with distinct biological properties: ‘regulated’ bone marrow adipocytes (rBMAds) and ‘constitutive’ bone marrow adipocytes (cBMAds), which were first defined in rodents (Scheller et al., 2015). rBMAds are present as individual adipocytes that are scattered throughout the BM, where they influence both hematopoiesis and bone homeostasis. cBMAds are clustered into confluent groups of BMAds. cBMAds are smaller than white adipocytes, but typically larger than rBMAds, that are situated in the distal skeleton. cBMAds and cBMAT are not biologically inert, because they are still altered in dietary and aging contexts. These cells are thought to participate less actively in the regulation of hematopoiesis or bone homeostasis (Fig. 2D) (Scheller et al., 2015), but there is insufficient experimental evidence to render definitive conclusions. The cBMAd subpopulation has remained largely uncharacterized in the human skeletal system and there is a major research opportunity for studies that analyze the heterogeneity, plasticity and physiological roles of BMAds at different skeletal sites. Due to the differences in BMAT subtypes and intrinsic cell types, it is very important that studies carefully consider and transparently report on the type of BMAT or the location of isolated BMAds for proper interpretation.

### 1.4 Pathological and genetic effects on BMAT, BMAds and bone marrow stromal cells (BMSCs)

The physiological responsiveness of BMAT to endocrine and/or metabolic cues is reflected by pathological responses to disease-related conditions. Skeletal inflammatory degenerative conditions (e.g., osteoarthritis, OA) alter the biological properties of BMAT by increasing the density of BMAds, based on studies using a mouse model for post-traumatic OA due to meniscectomy (Zapata-Linares et al., 2025). OA enhances the adipogenic and decreases the osteogenic potential of human MSCs derived from epiphyseal and metaphyseal marrow, while also modifying the transcriptomes of both BMAds and adipocytes from subcutaneous adipose tissue (SCAT) (Zapata-Linares et al., 2025). Furthermore, media conditioned by epiphysis specimens from OA patients secrete paracrine factors that reduce ALP activity and ECM mineralization in culture (Zapata-Linares et al., 2025). Hence, epiphyseal BMAT may be influenced by inflammatory processes in OA patients that compromise the ability of epiphyseal MSCs to support bone remodeling and repair. Similarly, OA also may alter the morphology of BMAds and their ability to produce glycerol and fatty acid substrates (lipolysis) that are required for the biological functions and metabolic activity of osteoblasts in osteochondral tissues of carpometacarpal and distal interphalangeal joints in the hand (Maniglio et al., 2025). Deep learning approaches combined with MRI data have been applied to understand the biological functions and pathophysiological activities of BMAT in a range of skeletal tissues, including the femoral head, total hip, femoral diaphysis, and spine of close to 50,000 participants in the UK Biobank. Genome Wide Association Studies (GWAS) meta-analyses combined with transcriptome-wide association studies revealed that between 40 and 100 unique genes may contribute genetically to the amount of BMAT present in each of the skeletal sites (Xu et al., 2025).

## 2 Overview of experimental approaches for isolation of BMAds or BMAT explants

### 2.1 Rationale for experimental guidelines to support BMAT research

BMAT is a fundamentally and biomedically important tissue in the context of several biological and pathophysiological conditions related to metabolism, bone diseases and cancer. In these research fields, various protocols for the isolation of BMAT or BMAds have been applied to address different biological questions. Several key studies have been summarized (Table 1) in which BMAT or BMAd samples were obtained from different animal species or human donors depending on the purpose of the study and the subsequent molecular analyses.

**Table 1:** Overview of key studies about experimental approaches for isolating BMAds or BMAT explants.

Year	Species	Skeletal site	Isolation technique	Research application	BMAd purity evaluation	Reference
1977	Rabbit	Sternum, thoracic vertebrae, femur, tibia, os calcis	Technique described in (Rodbell, 1964). BM is sliced into small pieces and placed in 10 ml of McCoy's medium in a 25 ml siliconized Erlenmeyer flask to which is added 10 mg of collagenase for each g of tissue. The mixture is incubated at 37 °C with slow constant gyratory rotation (50–100 rpm) for 1 h. Thereafter the contents of the flasks are gently stirred, the cells dispersed with a plastic spatula and any residual pieces of tissue are removed before centrifuging the suspension at 400 g. Fat cells and droplets of fat-ruptured cells float to the surface forming a layer which is decanted, resuspended in fresh medium and again centrifuged. The latter step is repeated, after which the cells were plated in a monolayer using 25 ml plastic culture flasks and incubated at 37 °C during which time the adipocytes adhere to the bottom of the flasks. After 4 h the medium was changed, and the cells were removed using EDTA and trypsin.	Lipid analysis	Microscopic examination of these preparations, stained with oil Red O, confirmed a pure preparation of BMAds containing lipid vacuoles	(Tavassoli et al., 1977)
1978	Rabbits	Tibia, femur	As described in (Tavassoli et al., 1977).	Adipocyte culture Cytochemistry	No	(Tavassoli, 1978)
2011	Mouse	Femur, tibia	Bones were cleaned and rinsed with 75 % ethanol and DEPC (diethyl pyrocarbonate) water to eliminate surrounding fat and muscle cells. Fresh BM were flushed out with PBS containing 1 % fatty acid-free BSA and 1 % RNAase and DNAase-free water using a 25-gauge needle from femurs and tibias. Red blood cells were lysed using red cell lysis buffer. After centrifugation at 3000 rpm for 5 min, floating adipocytes were isolated from BM stromal cells and then were washed with PBS buffer three times.	Microarray RT-qPCR Light microscopy Immunofluorescence microscopy	Immunofluorescence microscopy (Bodipy and Perilipin staining)	(Liu et al., 2011a)
2015	Mouse Rat	Tibia, femur, vertebrae	Tibial cBMAT (distal). Tibiae were removed and cleaned of muscle and tendon using gauze. A rotary power tool with a Dremel 545 Diamond cutting wheel was used to horizontally bisect the tibia at the base of the tibia/fibula junction. The distal portion was inverted into a 1.5-ml polypropylene microtube containing a hollow spacer and centrifuged at 3000 *g* to extrude the BM. The bone was removed and discarded, and the distal tibial BM was placed in warm KRH, pH 7.4 that had been pre-equilibrated overnight in an incubator at 37 °C, 5 % CO2 and re-pHed to 7.4. Washed adipose tissue pieces totaling 1 g were minced in 10 ml KRH containing 1 mg/ml collagenase type I and 3 % fatty-acid-free BSA in a 50-ml conical tube and placed in a shaking water bath at 100 r.p.m., 37 °C for 45–60 min. Digested tissue was pulled gently through a 10-ml polypropylene Luer-lock syringe (no needle) three times to complete disruption and then filtered through a 100-mm cell strainer into a fresh 50-ml polypropylene conical tube. Femur/tibia rBMAT. Femurs were isolated and cleaned, and the ends were removed with the rotary tool to expose the marrow cavity. The femurs and the proximal tibiae were inverted into 1.5 ml microtubes and centrifuged at 3000 *g* to separate the BM. The bones were discarded. Gentle pipetting was used to combine and resuspend the proximal BM in KRH containing 1 mg/ml collagenase and 3 % BSA in a 50 ml conical tube. The suspension was then incubated in a shaking water bath at 100 rpm, 37 °C for 15–20 min to liberate the rBMAT adipocytes. Vertebral cBMAT. The most proximal 10 tail vertebrae were separated and some of the surrounding muscle and tendon were removed with gauze. The vertebrae were added to a 50-ml conical tube with 2× the volume of KRH + 1 mg/ml collagenase and 3 % BSA. The tube was then incubated in a shaking water bath at 100 rpm, 37 °C for 20 min, with vigorous shaking by hand every 5 min to help dislodge remaining tissue on the outside of the vertebrae. After 20 min, the vertebrae solution was poured into a 10-cm dish. The vertebrae were quickly cleaned with gauze to remove any remaining soft tissue. Each vertebra was then bisected longitudinally with a diagonal cutter and put into a fresh 50 ml conical tube containing 2× the volume of KRH/collagenase/BSA solution. The bisected vertebrae were incubated in a shaking water bath at 100 rpm, 37 °C for an additional 20–30 min to liberate the cBMAT adipocytes. Vertebral rBMAT. Lumbar vertebrae were isolated and cleaned with gauze. The processing then continued as described for the vertebral cBMAT. Final processing for all adipocyte types. After filtration, the conical tubes were centrifuged at 400 *g* for 1 min to pellet the stromal vascular fraction and float the adipocytes. The pellet and the majority of the infranatant was carefully removed with a glass pipet and suction bulb. A plastic 1000 ml pipet tip was used to resuspend the adipocytes and transfer 300 ml of liquid containing 0.1–1.0 mg of cells to a 24-well plate-size transwell insert with 8 mm pores. Approximately 90 % of the liquid was removed by pressing the transwell membrane on a piece of dry paper towel. The cells in the insert were then washed twice in this manner with fresh KRH (no collagenase, no BSA). After the final wash and liquid depletion, the cells in the insert were collected in 300 ml of water and transferred immediately to a borosilicate glass tube for lipid extraction.	RT-qPCR Lipidomics	No	(Scheller et al., 2015)
2016	Rabbit	Humerus, tibia, and femur Radius and ulna’ epiphyses	Humeri, tibiae, and femurs were longitudinally bisected using a Dremel rotary tool with a 409-cutoff wheel, under a constant drip of sterile water was used during cutting to prevent overheating. BMAT was then removed using a stainless-steel spatula. Radii and ulnae epiphyses were removed by lateral incisions with the Dremel tool allowing access to the marrow cavity. BMAT was then extruded by first tracing the perimeter of the marrow cavity with a 2-in., 21-gauge needle, and subsequently scraping the BM out using a stainless-steel spatula.	RT-qPCR	No	(Cawthorn et al., 2016)
2017	Rat Mouse	Tibial distal epiphyses	BM was centrifuged into sterile isotonic saline and the adipocytes aspirated from the aqueous surface and pooled.	Lipid characterization (gas chromatography) Immunohistochemistry Immunogold electron microscopy	No	(Hopkins et al., 2017)
2017	Mouse	Femur, tibia	Long bones were collected and cleaned in sterile PBS. Both ends of femurs and tibias were snipped. Adapted from (Scheller et al., 2015; Liu et al., 2011a). Bones were placed in a 0.6-ml microcentrifuge tube that was cut open at the bottom and placed into a 1.5-ml microcentrifuge tube. Fresh BM was spun out by quick centrifugation (from 0 to 10,000 rpm, 9 s, room temperature). Red blood cells were lysed using RBC lysing buffer. After centrifugation (3000 rpm, 5 min, room temperature), floating adipocytes were collected from the top layer and washed with PBS three times.	RT-qPCR Flow cytometry (pre-adipocytes) TRAP technology	No	(Fan et al., 2017)
2018	Mouse	Long bones	BMAT were isolated from long bones by flushing the BM, quick high-speed spinning, and removing HSC (Liu et al., 2011b). Then the pellets containing the BMAT and HSCs were resuspended in PBS to let BMAT float on the top of the liquid suspension. HSC pellet was lysed with erythrocyte lysis buffer to remove red blood cells.	RT-qPCR	No	(Tencerova et al., 2018)
2019	Mouse	Long bones	As described in (Fan et al., 2017) A 0.6 ml microcentrifuge tube was cut open at the bottom and placed into a 1.5 ml microcentrifuge tube. Long bones were snipped on both ends and placed in the prepared 0.6 ml microcentrifuge tube. BM was flowed out by quick centrifugation (from 0 to 10,000 rpm, room temperature). Red blood cells from BM were lysed by ammonium-chloride‑potassium lysing buffer. After 3000 rpm centrifugation for 5 min at room temperature, floating adipocytes were collected as BM adipocytes from the top layer	RT-qPCR		(Zhang et al., 2019)
2019	Mouse Rat	Tibia, femur, vertebrae	As described in (Scheller et al., 2015)	RT-qPCR Lipidomics	No	(Scheller et al., 2019)
2019	Rabbit	Tibia, radius, ulna	As described in (Cawthorn et al., 2016)	Microarrays	No	(Craft et al., 2019)
2019	Rat	Caudal vertebrae	As described in (Scheller et al., 2015)	RT-qPCR	No	(Craft et al., 2019)
2020	Rabbit	Humerus, tibia, femur Radium and ulna epiphyses	As described in (Cawthorn et al., 2016) Tissue samples were immediately snap-frozen in liquid nitrogen for subsequent analysis.	RT-PCR Microarray analyses	No	(Suchacki et al., 2020)
2020	Rat	Proximal tibia	Tibia was cleaned of muscle and tendon using gauze and then cut axially at the tibia/fibula junction using a Dremel rotary tool with a Dremel 545 Diamond cutting wheel. The BM was removed by centrifugating at 3000 g for 1 min at 4 °C. The BM plugs from the distal tibia were bisected horizontally and the most distal, white portions pooled and used for protein extraction. Tissue was lysed at 4 °C on ice in SDS lysis buffer and homogenized by passing through a series of sequentially smaller needles.	Western blot RT-qPCR (as in Scheller et al., 2015)	No	(Suchacki et al., 2020)
2020	Rhesus macaques	Femur	BM adipose tissue was isolated from both femurs of adult male rhesus macaques as follows. BM tissue was gently disrupted using a 25-ml syringe loaded with a blunt needle, and the cell suspension was filtered through a 70-μm cell strainer. This suspension was centrifuged for 10 min at 300 *g* at room temperature, and the top adipocyte-containing layer was transferred to a tube containing 20 ml X-Vivo™ 10 (room temperature). The tissue was mixed by gentle inversion and left at room temperature for 15 min. For isolating the adipocyte-free fraction, the cell pellet was resuspended in 10 ml erythrocyte lysis buffer and incubated at 37 °C for 5 min. The cell suspension was diluted with 40 ml PBS and centrifuged for 10 min at room temperature. The resulting cell pellet was washed with PBS, resuspended in 1 ml PBS, and kept on ice. For isolating the adipocyte-bound fraction, the top adipocyte layer was transferred to a new tube containing 10 ml of collagenase solution (30 mg collagenase type II dissolved in 3.5 % BSA/PBS/2 mM CaCl2) and incubated at 37 °C for 40 min in a water bath. During incubation, the tube swirled by hand every five minutes and tissue lysis was monitored to ensure complete digestion.	Flow cytometry	No	(Robino et al., 2020)
2020	Rabbit Rat Mouse	Femur	As described in (Tencerova et al., 2018) The femurs were longitudinally bisected using a Dremel rotary tool with a 409 cutoff wheel, and the BMAT was then removed using a stainless-steel spatula.	Metabolomic analysis Lipid and sphingolipid analysis Western blot RT-qPCR Cell culture	No	(Zhang et al., 2020)
2021	Mouse	Femur, tibia	Femurs and tibiae were rapidly dissected into pre-warmed 37 °C HBSS buffer. After cutting the ends of the bones, whole BM was flushed into a 50 ml conical tube with a 10 ml syringe +22 gauge needle and resuspended into 20 ml fresh buffer +1 mg/ml collagenase. Marrow-depleted bones were placed into a separate tube in 20 ml buffer +1 mg/ml collagenase and finely minced to liberate any residual BMAds. Bone and BM preparations were centrifuged at room temperature, 400 *g* × 2 min, and BMAd-containing supernatant was decanted into a new tube prior to re-centrifugation at 400 *g* × 1 min. Infranatant and any residual pellet were removed using a pulled glass pipet until only 1–2 ml of liquid was remaining.	RT-qPCR	No	(Zhang et al., 2021)
2023	Mouse	Long bones	Adapted from (Fan et al., 2017) BM cells were directly flushed out by quick centrifuge (from 0 to 9400 *g*, approximately 15 s at room temperature) after cutting both ends of long bones, then resuspended by PBS and filtered through 70 μm cell strainer. The cells were spun down at 500 *g* for 5 min at room temperature. The floating mature lipid-laden adipocytes were collected from the top layer and washed with PBS for three times.	Single-cell RNA-seq RT-qPCR Flow cytometry	No	(Inoue et al., 2023)
2006	Human	Posterior iliac crest	Collection of 10–20 ml of marrow during BM aspiration (standard protocol) 10 ml of diluted BM aspirate was centrifuged at 550 *g*; the supernatant was further centrifuged at 475 *g* for 10 min. All but the erythrocyte-rich pellet was transferred to a fresh 50 ml conical tube and centrifuged for 5 additional minutes at 475 *g*. The supernatant was then transferred to a T-25 flask, which was filled to the rim with adipocyte maintenance medium and inverted for incubation. After approximately 2 days in culture, the adherent adipocytes were rinsed, and the flask was filled to the rim with fresh adipocyte maintenance medium. This method of culturing low-density cells on the upper surface of a flask, known as ceiling culture, was adapted from (Sugihara et al., 1986). Medium in ceiling cultures was changed every 2 weeks.	RT-qPCR Culture	No	(Mackay et al., 2006)
2015	Human	Femoral head	Collection during hip-replacement surgery Femoral heads were placed in physiological saline, and placed into a Pyrex dish cancellous bone fragments measuring ∼3 to 5 mm2 were dissected from the shaft using a surgical Rongeur. BM was isolated from cancellous bone tissues by flushing individual fragments with 10 ml volumes of PBS expelled from a syringe fitted with a 25-gauge needle and filtering the effluent through 70 μm strainers seated into 50 ml conical tubes. The resulting BM cell suspensions were centrifuged for 3 min at room temperature at 300 g. Pellets were resuspended in ROCK medium and transferred to 24-well tissue culture plates.	Explant culture Co-culture with cancer cell lines Migration assays	No	(Templeton et al., 2015)
2016	Human	Femur	Collection of 2–3 ml of BM during surgery for total knee arthroplasty Samples were processed according to (Mackay et al., 2006).	RT-qPCR	No	(Chen et al., 2017)
2018	Human	Femoral head	Collection during hip-replacement surgery Each femoral head was cut into four parts. After prompt washing in DMEM, any visible blood vessels were removed and the tissue was minced into smaller pieces. Bone was treated with a solution containing 1 mg/ml of type I collagenase and 1 % human albumin (Albital, Kedrion, Lucca, Italy) at 37 °C for 90 min. After collagenase digestion, samples were filtered through a 200 μm nylon sieve to remove stromal elements. Cells were then washed four times with DMEM and centrifuged at 250 *g* for 5 min. Collection of the floating layer after each centrifugation provided a pure fraction of floating adipocytes and a pellet containing stromal cells.	Immunofluorescence Culture Microarrays	After the last centrifugation, the purity of isolated cells was confirmed by immunofluorescence staining with Nile Red.	(Mattiucci et al., 2018)
2019	Human	Femoral head	Collection during hip-replacement surgery BM biopsies were fragmented, washed once with complete RPMI medium and treated with purified collagenase (20 U/ml in complete RPMI medium) for 1 h at 37 °C. After centrifugation for 10 min at 150 *g* with low break and before Ficoll purification, adipocytes formed a floating layer on the top of the medium which was carefully transferred into a fresh 15 ml tube. Tubes containing adipocytes were centrifuged at 150 *g* for 10 min and afterwards, adipocytes were isolated by flotation.	Microarrays Flow cytometry RT-qPCR ROS measurement	No	(Miggitsch et al., 2019)
2020	Human	Femur	Collection during hip-replacement surgery For microarrays, as described in (Mattiucci et al., 2018). After surgical isolation, tissues were washed and stored in ice-cold DPBS for transport to a sterile tissue culture hood. Therein, DPBS was decanted through a sterile 300 μm nylon filter to remove blood, lipid and small debris. The remaining washed tissue was then transferred to a sterile, pre-weighed petri dish (100 mm) and tissue mass recorded. A solution of collagenase type I was made at 1 mg/ml in KRH buffer pre-warmed to 37 °C; sufficient volume was made to allow for 2 ml per mg tissue and the solution was passed through a 0.22 μm filter before use. After weighing, each tissue was minced in the petri dish using a sterile scalpel and scissors, then transferred to a Falcon tube containing the collagenase solution. Tissues in collagenase were then incubated for 45 min in a shaking water bath (120 rpm) at 37 °C. Next, collagenase-digested tissue was passed through a 300 μm nylon filter and the cells within the filtrate were washed with fresh KRH buffer. Samples were then centrifuged at 500 *g* for 5 min at 4 °C. The floating adipocyte layer was transferred by pipette to a new tube.	Microarrays RT-qPCR	Histological analysis to confirm the presence of BMAds.	(Suchacki et al., 2020)
2020	Human	Femoral cavity (proximal metaphysis and diaphysis)	As described in (Attane et al., 2021).	Immunofluorescence Proteomic Lipidomic Metabolic assay	Immunofluorescence microscopy (Bodipy, DAPI and Perilipin staining).	(Attane et al., 2020)
2021	Human	Posterior iliac crest	Collection during BM aspiration (standard protocol), collected in EDTA tube. 5 ml of PBS was added and spun at 377 *g* for 8 min at 4 °C. The top layer was transferred to a microfuge tube and trizol reagent was added (1:1) and the samples were stored at −80 °C. Samples in the tiger-top tubes were spun at 2054 *g* for 10 min at 4 °C. The top layer was aliquoted and stored at −80 °C.	RT-qPCR Samples were studied using RNA-seq in (Aaron et al., 2021)	No	(Fazeli et al., 2021)
2021	Human	Femoral cavity (proximal metaphysis and diaphysis)	Collection by aspiration during hip-replacement surgery with 60 ml syringe and a soft canula. Collect 10–20 ml and place it in the 37 °C pre-warmed tube containing 20 ml KRBHA. Place a 100 μm cell strainer on top of a 50 ml tube, rinse the strainer with KRBHA, and pour the tissue sample on top. Pour 20 ml KRBHA on the sample to remove blood. Collect BM sample present on the strainer with tweezers and transfer it to a petri dish. Using tweezers and scissors isolate the areas of BMAT that are recognized by their yellow color and transfer this material to a new petri dish. Add 10 ml KRBHA to the petri dish to wash the BMAT. Transfer BMAT to a pre-weighed 14 ml tube. Weigh the tube containing BMAT and calculate the tissue weight. Use 2.5 ml collagenase 1× solution per gram of tissue. Calculate the volume of collagenase 1× solution necessary for the experiment and prepare it by diluting the 5X stock solution in PBS 2 % BSA. Add the collagenase 1× solution to the tube containing the BMAT, close the tube and seal it with parafilm. Place the tube in a horizontal position on an orbital shaker at 37 °C, 150 rpm. Incubate for roughly 20 min. Place a 100 μm cell strainer on top of a 50 ml tube, rinse the strainer with KRBHA, and filter the suspension through it to remove cellular debris, undigested fragments, and bone trabeculae. Pour 20 ml KRBHA on the strainer to recover a maximum of BM-Ads. Wait 1–3 min to allow the lipid-laden adipocytes to rise up and float at the surface Remove 15–18 ml KRBHA from under the floating BM-Ads with a syringe and a 21G needle to facilitate the collection of the adipocytes and their transfer to a new tube - Collect gently floating BMAds with a 1 ml pipet with standard P1000 pipette tip and transfer them to a 2 ml tubes. Wash adipocytes by repeating the following steps 3 times: add 1 ml KRBHA and mix the suspension gently by tilting; wait until adipocyte rise again to form an upper layer, KRBHA being in the lower layer; using a 2 ml syringe with a 21G needle, aspirate the KRBHA and discard it. Add 1 ml KRBHA and wait until adipocytes rise again to form an upper layer. Centrifuge for 5 min at 200 *g*, 22 °C. This step allows to pellet contaminant cells. Transfer floating adipocytes to a new tube. At this step, BMAds can be used for cell culture and functional experiments or can be frozen. Preparation of adipocytes for freezing. Freezing leads to adipocyte lysis. Thus, for functional assays, culture or imaging, adipocytes must be used directly after isolation and washing steps. For molecular analyses (RNA, protein, lipid content), we recommend freezing a known volume of adipocytes (without KRBHA) in liquid nitrogen and storing these samples at −80 °C until use. Make several aliquots when possible.		Keep 30 μl of BMAds in a separate 2 ml tube to validate the purity of the cell population. Confocal microscopy (BODIPY 493/503).	(Attane et al., 2021)
2025	Human	Femoral head (epiphysis) & metaphysis	Human femoral head, metaphyseal bone and subcutaneous AT from the gluteus-femoral zone (SCAT) are obtained from patients undergoing hip replacement surgery. Adipocytes and MSCs are harvested from the bone marrow portion of the femoral head and metaphysis and from the SCAT of OA patients. Tissues are minced into small pieces and treated with collagenase A (1 mg/ml) at 37 °C for 90 min on agitation. Samples are then filtered through a 100 μm nylon, washed three times with DMEM and centrifuged at 150 *g* for 5 min. Collection of the floating layer after each centrifugation provides a pure fraction of floating adipocytes that were directly used for total RNA isolation.	Histology RNA-seq	As described in (Attane et al., 2021).	(Zapata-Linares et al., 2025)

**Abbreviations:** BM, bone marrow; BMAd, bone marrow adipocytes; BSA, bovine serum albumin; cBMAT, constitutive bone marrow adipose tissue; DPBS, Dulbecco's phosphate-buffered saline; EDTA, ethylenediaminetetraacetic acid; HBSS, Hanks' balanced salt solution; HSC, hematopoietic stem cells; KRBHA, Krebs ringer buffer HEPES albumin; KRH, Krebs-Ringer solution HEPES-buffered; PBS, phosphate-buffered saline; RBC, red blood cells; rBMAT, regulated bone marrow adipose tissue; RT-qPCR, reverse transcription-quantitative polymerase chain reaction; TRAP, translating ribosome affinity purification.

BMAT is a heterogeneous tissue based on its source, location and method of isolation, and this heterogeneity will qualify the interpretation of results (Lucas et al., 2021). Therefore, rigorous application of BMAT isolation protocols requires documentation of essential details and strict adherence to common standards to ensure the reproducibility of data and uniformity of samples to enable follow-up studies, replication by other research groups and future collaborations. While the application of common standards cannot be made mandatory across all studies (e.g., for historical, budgetary and/or legal reasons), this paper provides recommendations that reflect the consensus of BMAS-affiliated authors who trust that voluntary adherence to these guidelines will improve transparency, scientific rigor and data interpretation.

This current narrative provides an update on how the field has evolved since protocols for the isolation of human or mouse BMAT were last evaluated and formalized in previous BMAS guidelines (Lucas et al., 2021; Tratwal et al., 2020). Beyond this initial standardized set of experimental procedures for BMAT isolation, recent papers including three recent informative studies (Xu et al., 2025; Maniglio et al., 2025) have advanced additional new BMAT isolation protocols and techniques, including BMAd explants and multi-omics approaches (e.g., transcriptomics, proteomics, metabolomics, lipidomics) and deep learning. Collectively, these experimental strategies and the resulting multidimensional data sets bring new perspectives to the formation and function BMAT, as well as the biological roles of BMAT in bone homeostasis, overall physiology, systemic metabolism, and pathological contexts.

### 2.2 Strategies for BMAT explant or BMAd isolation depending on the species and skeletal site

The choice of the skeletal site depends mostly on the animal species and the specific type of BMAds that are targeted by the study. Many studies use rabbits and rats as animal models, because they are larger in size compared to laboratory mice. Early studies in the field focused on rabbit BMAT for ease of study, while mice and rats represent versatile experimental models that have become more commonly used in recent studies. Rabbits have been a particularly relevant model for BMAT biology in humans, because their bone anatomy is more similar to humans (e.g., presence of haversian canals) and rabbits develop extensive intramedullary BMAT (Pazzaglia et al., 1987). In rabbits, the femurs, tibiae, radii and ulnae are typical anatomical locations for isolation of regulated BMAT (Fig. 2C) (Cawthorn et al., 2014; Cawthorn et al., 2016), while calcaneus bone is useful for the isolation of constitutive BMAT. Typically, a rotary cutting tool (e.g., Dremel) is used to remove the extremities of long bones and to bisect the bones longitudinally to expose BM. In rats and mice, femurs and proximal tibiae represent sites for isolation of rBMAT, while distal tibiae and caudal vertebrae are sites for harvesting cBMAT. The small size of the bone segments in rabbits, rats and mice allows for quicker isolation of BMAT compared to larger animals. In principle, the experimental trade-off is that the smaller the animal model, the quicker the harvesting procedure, but the lower the specimen yield. In rodents, BMAT can be obtained by flushing the marrow with a syringe or by extruding the marrow through centrifugation of the segment in microfuge tubes with a hollow spacer. However, the limited yield of adipocytes from small rodents, especially mice, requires pooling of samples from at least two animals to produce enough material for subsequent experiments. Protocols developed for various animal models (including mice, rats, rabbits, guinea pigs, pigs, cows, dogs, horses and monkeys) may differ because the relative amount of BMAT differs among species (Scheller et al., 2015).

Current isolation techniques for BMAT/BMAds have in common that they are either based on collagenase or on a quick centrifugation method (details presented in Table 1). BMAT isolation protocols using rats and rabbits generally apply collagenase-based procedures for cell isolation because the composition and input amount of BMAT is sufficient to permit enzymatic digestion with sufficient yield of BMAds (Scheller et al., 2015; Suchacki et al., 2020; Tavassoli et al., 1977; Craft et al., 2018; Cawthorn et al., 2016; Zhang et al., 2021; Scheller et al., 2019; Hopkins et al., 2017) (Table 1). Given the many different anatomical locations from which BMAT is isolated, the variability in composition and yield, and the different isolation methods, it is highly recommended to document and report sufficient detail about the isolation procedure in relation to the skeletal site.

### 2.3 Isolation of BMAds from mammalian models

In large mammalian models (e.g., pig, cow, dog, and monkey), bone segments from femurs and tibiae are the preferred skeletal sites for isolation of BMAT samples (Scheller et al., 2015). In rhesus macaques, BMAds have been isolated from the femoral BM by disrupting this tissue using a syringe loaded with a blunt needle. The resulting cell suspension was filtered through a 100 μm cell strainer, then layered on a Ficoll density gradient prior to centrifugation. In the final step, the BMAd-enriched fraction was collected as the floating cell suspension at the top of the gradient (Robino et al., 2020). The greater amount of starting material available from larger species typically permits collection of sufficient cell quantities for analysis.

BMAT/BMAd isolation procedures using mouse BM typically yield insufficient amounts of cells for many experiments (Scheller et al., 2015). One of the earlier studies in the field collected BMAds from yellow BM harvested from larger rodents (i.e., guinea pigs) by longitudinal incision of tibiae (Zakaria and Shafrir, 1967). Several studies have addressed the challenge of low BMAT/BMAd yields by applying methods that rapidly flush marrow upon removal of the ends of long bones (i.e., epiphyseal region) by either centrifugation or pressure applied through a syringe with a needle. The resulting cell suspension is then centrifuged, or allowed to separate based on differences in cell buoyancy under normal gravity, to isolate the top layer as the BMAd layer (i.e., collection of ‘floating cells’). In some cases, red blood cell (RBC) lysis of the BM sample is used to eliminate intact erythrocytes and the top layer of cell suspension can be rinsed with phosphate-buffered saline solution (e.g., PBS) to improve the yield of BMAds (Tencerova et al., 2018; Fan et al., 2017; Inoue et al., 2023; Liu et al., 2011a; Zhang et al., 2019).

One variant of this method collects BMAds from the flushed-out BM, but also from minced residual bone that can be further extracted using collagenase (Zhang et al., 2021). While this procedure may increase cell yield, it may also compromise relative cell purity. Another variant uses BM plugs from distal tibiae that are highly enriched in BMAT (Li et al., 2022a); the enrichment of BMAT in these distal tibia plugs permits rapid isolation of degradation-sensitive polyadenylated RNAs for bulk RNA-seq. A third protocol considers age-related differences in the purification of BMAds isolated from young and mature mice (i.e., at 2 vs 6 months), and applies radiation to increase the very low abundance of BMAds in young mice (Hirakawa et al., 2023). Because radiation has pleiotropic effects, its application to boost cell yields may have unintended biological consequences.

### 2.4 Isolation of BMAds from clinical specimens

BMAT collection from patients is mainly performed from skeletal sites of appendicular bones that are collected as clinical waste during orthopedic surgery (e.g., hip and knee arthroplasty). Hip replacement allows for the collection of higher amounts of BMAT from the femoral proximal epiphysis and metaphysis, which are sites of yellow marrow in adults. Knee arthroplasty permits the collection of BMAT from femurs and tibiae, specifically from the respective mid-diaphyseal region that is rich in yellow marrow compared to the epi- and metaphyseal regions (Fig. 2A). While yellow BM is present, other studies indicate that the medullary space of the proximal epiphysis and diaphysis contain a mix of red and yellow BM (Attane et al., 2021). BMAds can be obtained from these BMAT sources but also from standard human BM aspirates derived from hip bone (i.e., iliac crest).

BMAT isolation methods for humans depend on the type of collection (BM aspirate vs surgery) and skeletal site (e.g., femur, hip, iliac crest). Different methods have been developed in which BMAd isolation is achieved with or without collagenase digestion, depending on subsequent molecular analyses. BM aspirates are effective because they contain sufficient quantities of BMAT. A number of published papers examined BMAds in BM aspirates from femoral diaphysis (Suchacki et al., 2020; Attane et al., 2020), trabecular bone of the proximal femoral metaphysis/epiphysis (Suchacki et al., 2020), femoral head (Mattiucci et al., 2018), femoral BM fluid (Crissman et al., 1979), proximal tibia (Griffith et al., 2009a), iliac crest (Poloni et al., 2013; Whitney et al., 2021; Whitney et al., 2020), or vertebral bodies (Whitney et al., 2021; Whitney et al., 2020)(Table 1).

BM aspirate samples are usually processed by separation-based centrifugation without the use of collagenase (Aaron et al., 2021; Fazeli et al., 2021; Chen et al., 2017). BM specimens obtained during orthopedic surgery are typically subjected to collagenase digestion, to optimize the quantity and quality (i.e., BMAd purity) of human BMAds for subsequent analyses (Suchacki et al., 2020; Attane et al., 2020; Miggitsch et al., 2019). Notably, humans BMAds isolated from femoral epiphysis versus metaphysis are divergent (Zapata-Linares et al., 2025). Furthermore, collagenase digestion has been used to directly examine extramedullary adipocytes that cannot be obtained by other currently available procedures.

### 2.5 Adipocytes obtained by ex vivo differentiation of BMSCs

Another approach for studying BMAd function and metabolism is via isolation of BMSCs and differentiation of the cultured cells ex vivo towards the adipogenic lineage. Protocols for isolation of human BMSCs use BM aspirates and purification by Ficoll gradient followed by expansion of the BMSC layer in culture (Lucas et al., 2021). BMSCs are isolated from rodent bones by flushing and digestion of bone fragments with collagenase (Tratwal et al., 2020).

In general terms, current isolation protocols exhibit similarities and differences that reflect the diversity of the source materials. Typically, protocols are adapted to optimize BMAd yields for cell culture or the integrity of biomolecules (e.g., mRNA, protein, metabolites) in tissue or cell lysates in subsequent biochemical analyses. Thus far, there are not many animal or human studies that attempt to distinguish rBMAds versus cBMAds derived from BMSCs (Scheller et al., 2015; Craft et al., 2018; Zhang et al., 2021; Li et al., 2022b; Tratwal et al., 2022). It remains unclear if site-specific BMAd differences are maintained following differentiation ex vivo. Differentiation of BMSCs with thiazolidinediones that potently stimulate adipogenesis by activating the nuclear receptor master regulator Peroxisome Proliferator Activated Receptor Gamma (PPARγ/PPARG) typically induce an overt white adipocyte phenotype ex vivo that would obliterate any subtle cell type differences that may exist in vivo. Moving forward, future protocols should provide information on input sample amounts, cell yields and whether it was necessary to pool BMAd samples to permit standardization, optimization and quality control of adipogenic differentiation of BMSCs.

## 3 Criteria for optimal BMAd or BMAT isolation

Because BMA is highly variable based on histochemical analyses (Tavassoli et al., 1977), magnetic resonance imaging (MRI) and microcomputed tomography (μCT) (Scheller et al., 2015), it is important to account for the species, age, sex, pathophysiological condition and anatomical site. Rigorous reporting on BMAT and BMAds requires description of several principal criteria, including the precise skeletal location, presence of adipocytes and the potential presence of other BM cells.

### 3.1 Current strategies for validation of BMAT explant isolation

Macroscopic observation (Attane et al., 2020; Templeton et al., 2015) can help with adjusting the isolation protocol and to estimate the amount of adiposity in large human samples (e.g., yellow versus red BM). Histology of specimens using classical hematoxylin and eosin staining is also recommended for BMAT analyses. Regions of interest in bone that contain relatively pure yellow BM or cBMAds within whole BMAT can be easily identified in distal tibiae, calcaneus or caudal vertebrae. This yellow BM can be distinguished from samples with red BM that are depleted of adipocytes and mainly contain interspersed hematopoietic cells, as is observed in femur and proximal tibia or lumbar vertebrae (Suchacki et al., 2020; Tavassoli et al., 1977; Cawthorn et al., 2014). Analysis of the density and size of BMAds in situ permits assessment of heterogeneity in adipocyte content and morphology (Cawthorn et al., 2014; Templeton et al., 2015; Dello Spedale et al., 2022). While these parameters are not routinely determined, it is recommended that this analysis is included in experimental protocols and reported according to appropriate guidelines (Bravenboer et al., 2019).

The analysis of adipocyte parameters is facilitated by the robust positive correlation between adiposity and detection of adipocyte biomarker proteins like adiponectin (ADIPOQ) and perilipin 1 (PLIN1). Their status as highly specific fat markers were recently further validated by RNA-seq analysis of multiple musculoskeletal tissues (Thaler et al., 2022). Because PLIN1 expression is specific for mature adipocytes, detection of PLIN1 protein has been used to estimate the degree of adiposity in BMAT in different species (Cawthorn et al., 2014). Transcriptomic analyses that compared different depots of WAT with BMAT in rabbits successfully permitted discrimination of the capacity for energy metabolism in each fat pad (Suchacki et al., 2020; Craft et al., 2019).

Comparison of the fat, DNA and protein content of tibial yellow BM and epididymal adipose tissue in guinea pigs provided an early indication of the similarities in these two types of fat depots and their adipocyte content (Zakaria and Shafrir, 1967). Because BMAT results are species-dependent, it is unclear whether these guinea pig data are relevant to outcomes for other rodents, such as rats and mice. Recent mouse studies found differences in total lipid content in femur versus tibia BM: femoral BM contains less lipid than tibial BM, while the latter contains much less lipid than WAT (Bartelt et al., 2017).

In addition to histology and molecular analysis, experiments assessing BMAT viability or functionality could be considered to validate the quality of isolated BMAT. One study assessed the viability of BMAT and WAT explants by measuring release of lamin A/C (LMNA). Because LMNA is a non-secreted protein that is an integral component of the nuclear envelope, its release into conditioned media provides a compelling marker of cell lysis during explant culture (Cawthorn et al., 2014). Others have assessed the viability of WAT explants by measuring the release of lactate dehydrogenase LDH (Du et al., 2011) or using calcein-acetoxymethyl (Calcein-AM) staining (Schopow et al., 2020). Hence, these methods would also be useful for future BMAT explant studies.

Functional experiments with yellow BMAT and WAT in guinea pigs suggested that these tissues have similar glucose and fatty acid (FA) metabolism (i.e., de novo lipogenesis, FA use and release) (Zakaria and Shafrir, 1967). More-recent studies reported low or no lipolytic activity in BMAT from dogs (Tran et al., 1981), or BMAT from humans compared to subcutaneous white adipose tissue (SCAT) (Attane et al., 2020). Thus, assessing lipolytic activity as a functional criterion for BMAT may be informative, but does not provide a definitive marker.

Secretion of adiponectin reflects the endocrine function of BMAT (Cawthorn et al., 2014), (Attane et al., 2020). Catecholamines may increase intracellular cAMP levels in BMAT and SCAT, which reflects the physiological responsiveness of BMAT (Attane et al., 2020), while catecholamines increase adiponectin release from visceral adipocytes (Musovic and Olofsson, 2019). It remains uncertain whether catecholamine increases adiponectin secretion in BMAT, but measurements of modulations in cAMP production (e.g., in response to catecholamines) and subsequent adiponectin release would provide a robust functional outcome that should be considered for assessing the functional activity of BMAT explants.

### 3.2 Adipose biomarkers for the characterization of BMAds

Regardless of the selected isolation method (with or without collagenase), many studies compare BMAds with extramedullary adipocytes from different visceral or subcutaneous WAT depots. These comparisons analyze mRNA or protein expression of typical adipocyte markers, including ADIPOQ, PLIN1, PPARG, and leptin (LEP), as discussed previously (Lucas et al., 2021). Recent RNA-seq data validated the corresponding mRNAs and generated an expanded set of genes, designated the Fat76 gene set, that encompasses additional mRNA biomarkers for fat tissue (Thaler et al., 2022). The first transcriptomic analysis of BMAds isolated from femurs and tibiae of aging male mice indicated that several typical adipocyte-specific genes (e.g., PLIN1, ADIPOQ, LEP) are expressed at lower levels in BMAds when compared to extramedullary adipocytes (Liu et al., 2011a). This profile has been confirmed by most subsequent studies performed in rats, mice, and humans, irrespective of skeletal location or referent adipose depot, and was also found in the complete BMAT of rabbits (Table 2). Although BMAds can appear to be at an early stage of differentiation both in animal models (Liu et al., 2011a) and human samples (Miggitsch et al., 2019), the expression of these genes is still considered specific to the adipocyte lineage, notably in comparison to other concomitantly isolated BM cells (Scheller et al., 2015; Suchacki et al., 2020; Liu et al., 2011a; Mattiucci et al., 2018). *PPARG* appears to be the least downregulated of the adipocyte genes in BMAds versus other fat depots. This finding is expected because many lineage-specific gene regulators (e.g., *PPARG, RUNX2, SOX9*) (Rosen et al., 2009; van Wijnen et al., 2004; Lefebvre et al., 2019) are also broadly expressed in non-committed mesenchymal stromal cells (Dudakovic et al., 2014; van de Peppel et al., 2017), and *PPARG* also is also expressed in macrophages and lymphocytes (Goyal et al., 2018).

**Table 2:** Range of values obtained during characterization of BMAds or BMAT compared to referent adipocytes or whole adipose tissue using gene expression methods.

				Adipocytes markers expressed as a ratio compared to referent adipocytes				Other BM cell markers		
Species / model	Referent adipocytes: localization	Isolated BMAT: localization	Analysis technique	PPARG	ADIPOQ	LEP	PLIN1	Immune cell markers	Others	Reference

BMAT explants										
Rabbit (New Zealand), males	Gonadal and inguinal whole adipose tissues	proximal tibia, distal tibia, radius/ulna	Micro-array (Affymetrix)			From 0.13 to 0.39 (vs gonadal) or 0.09 to 0.32 (vs inguinal) *^,^⁎⁎				(Craft et al., 2019)
Rabbit (New Zealand), males	Gonadal and inguinal whole adipose tissues	proximal tibia, distal tibia, radius/ulna	Micro-array (Affymetrix)		From 0.15 to 0.59 (vs gonadal) or 0.17 to 0.65 (vs inguinal) ⁎⁎	From 0.24 to 0.32 (vs gonadal) or 0.20 to 0.26 (vs inguinal) ⁎⁎				(Suchacki et al., 2020)

Isolated BMAds										
Species / model	Referent adipocytes: localization & method	Isolated BMAds: localization & method	Analysis Technique	PPARG	ADIPOQ	LEP	PLIN1	Immune cell markers	Others	Reference
Mouse (C57Bl/6 J), males	Epididymal adipocytes using collagenase	Femur and tibia, by flotation	qPCR analysis (ref gene 18S)	ND	∼0.27	∼0.37	∼0.31	Immunostaining with CD11b antibody		(Liu et al., 2011a)
			Microarray (GeneChip Mouse Gene 1.0 ST Array platform, Affymetrix)	From 0.097 to 0.169⁎	From 0.024 to 0.038⁎	From 0.002 to 0.007⁎	From 0.009 to 0.012*			
Rat (Sprague Dawley), males & females	Subcutaneous inguinal adipocytes, using collagenase	Femur and proximal tibia (rBMAd) and cBMAd, using collagenase	qPCR (ref gene TBP)	From 1 to 3.2 ⁎⁎⁎	ND	ND	ND			(Scheller et al., 2015)
Mouse (C57BL6J), males	Epididymal adipocytes with collagenase	Tibias and femurs, by flotation and RBC lysis	qPCR (ref genes 36B4 HPRT)	∼0.022	∼0.009	∼0.007				(Tencerova et al., 2018)
Human, males & females, mean age 67 yrs. for SCAT & 78 yrs. for BMAd).	Abdominal subcutaneous adipocytes, with collagenase	Femoral head with collagenase	MicroArray (Human GeneChip® HTA 2.0 Arrays, Affymetrix)	∼0.59	∼0.59	∼0.41	∼0.61	CD11B: ∼1.17 F4/80: ∼1.19 CD13: ∼1.40 CD14: ∼1.03 compared to referent adipocytes	Osteoblast markers: RUNX2: ∼1.20 ALPL: ∼ 1.16 compared to referent adipocytes	(Mattiucci et al., 2018)
Human, males & females, mean age 65 ± 13 yrs.	Hip subcutaneous adipocytes, with collagenase	Femoral head, using collagenase and further process to remove hematopoietic and stromal cells	qPCR (18 s)	∼0.33	∼0.038	ND	ND	Flow cytometry using CD45 antibody	Flow cytometry (CD31 vs CD105 & CD24: endothelial vs MSCs	(Miggitsch et al., 2019)
			Microarray (HuGene 2.1, Affymetrix)	At least <0.5						
Human, males & females, 67.1 ± 5.9 yrs	Gluteofemoral subcutaneous adipocytes, using collagenase	Proximal femoral diaphysis or proximal femoral trabecular metaphysis, with collagenase	qPCR analysis (ref genes IPO8 or RNA18SN5)	0.034 to 0.047⁎⁎	0.021 to 0.037⁎⁎			CD45 and CD11b less expressed (at least 4.8 and 3.8 times, respectively) compared to the respective isolated other BM cells.		(Suchacki et al., 2020)
Human, males & females, median 70 years (range 25–84)	Gluteofemoral subcutaneous adipocytes, using collagenase	Femoral epiphysis and metaphysis, with collagenase	RNA-seq	0.388 (meta) to 0.487 (*epi*)	0.314 (meta) to 0.418 (epi)	0.935 (epi) to 1.731 (meta)	0.263 (meta) to 0.348 (epi)			(Zapata-Linares et al., 2025)

Results are assessed using published values of mRNA expression levels of each gene by calculating the ratio of expression level in BMAds / expression level in referent adipocytes; ND not done.

⁎ according to age.

⁎⁎ according to BMAT site.

⁎⁎⁎ according to adipocyte type (rBMAd, cBMAd) and to sex.

Adiponectin is the most prominent adipokine in circulation and is typically expressed higher in adipogenic cells compared to other non-adipogenic mesenchymal cell types. Nevertheless, *ADIPOQ* mRNA levels in BMAds are lower than in white adipocytes and exhibit considerable variability between studies, with relative expression ranging from ∼0.009 to 0.27 in rodents and ∼ 0.038 to 0.59 in humans. Isolated human BMAds express similar protein levels of ADIPOQ and LEP based on proteomics (Attane et al., 2020) or ELISA measurements in conditioned media (Miggitsch et al., 2019). Notably, standardization of analytical methods (Table 2) and systematic assessment of cell contamination in adipocyte preparations (which can introduce bias, as discussed below) are still lacking. As the field progresses, it is recommended that future studies establish a reference range for mRNA expression of adipocytic marker genes in BMAds compared to a reference range for white adipocytes. A standard immature fibroblast cell type (e.g.,BMSC) could function as a baseline. Quantitative analysis of mRNA markers would generate quality control parameters to define what constitutes a ‘good isolation procedure’.

To validate the relative purity of isolated BMAd preparations, other strategies encompass staining of isolated cells for nuclei using the blue fluorescent dye 4′,6-diamidino-2-phenylindole (DAPI) and neutral lipids using boron dipyrromethene (BODIPY) or the lipophilic stain Nile Red to analyze the potential presence of contaminant cells (Suchacki et al., 2020; Attane et al., 2021; Lucas et al., 2021). Immunostaining of isolated cells with macrophage marker (CD11b) has also been performed for mouse (Fan et al., 2017; Liu et al., 2011a) and human (Miggitsch et al., 2019) samples with concomitant staining for lipids or PLIN1. Contamination of non-adipocyte cells in BMAd samples was less than 2 % when using flow cytometry or 10–17 % when using microscopy to quantitate CD11b-positive cells. Alternatively, gene expression of markers for leukocytes (CD45/PTPRC) and macrophages (CD11b/ITGAM) can be performed and compared to other isolated BM cells (Suchacki et al., 2020; Mattiucci et al., 2018). The expression of osteoblast biomarkers has also been analyzed to detect impurities in BMAd preparations (Mattiucci et al., 2018) (Table 2). The presence of adipocyte progenitors as contaminants in BMAd preparations has not yet been addressed but coudl be solved by developing markers based on high-throughput single-cell RNA-sequencing and multispectral microscopic imaging. BMAd viability should be checked to validate the isolation method, as previously performed for subcutaneous adipocytes using calcein-AM and propidium iodide staining (Harms et al., 2019) or with the Alamar blue method (Urbonas et al., 2023). These strategies are very useful but are not typically reported for BMAds.

Although collagenase is often applied to improve BMAd yield, the use of collagenase can lead to significant adipocyte lysis: a study of femoral BMAds from rhesus macaques showed more than 50 % loss of viable cells after 48 h in culture (Robino et al., 2020). As discussed for BMAT, functionality is also an important criterion for BMAds. As lipolytic activity is low in BMAds compared to subcutaneous adipocytes (Scheller et al., 2019; Attane et al., 2020), the analysis of adiponectin or leptin secretion by human isolated BMAds after 48 h in culture (Miggitsch et al., 2019) may represent an effective strategy to evaluate their functionality and account for inter-patient variability.

Current distinctions between cBMAds and rBMAds are primarily based on skeletal site (Scheller et al., 2015). Indeed, BM from rBMAT (femur, proximal tibia and lumbar vertebrae) or cBMAT (distal tibiae, caudal vertebrae) are separately digested to isolate rBMAds and cBMAds, respectively. The cBMAds have increased unsaturated FAs compared to rBMAds (Fig. 2D), suggesting that differences in lipid composition and/or FA desaturase enzyme expression can be used as a criterion to validate the isolation of these two types of adipocytes. In conclusion, the definition of what constitutes high quality BMAT or BMAds preparation requires further development of effective criteria. The following section will discuss current challenges and recommendations.

### 3.3 Limitations of current experimental strategies

#### 3.3.1 BMAT-related sample pooling

The number of BMAds and amounts of other BMAT-derived biological material that can be analyzed is often limited, especially for murine BMAds. The low cell yields necessitate pooling of samples from multiple animals and/or different bone types (Scheller et al., 2019; Tencerova et al., 2018; Liu et al., 2011a). The type and number of samples that are combined for analysis should be transparently reported to allow a better understanding of the data.

#### 3.3.2 Definition of the cellular composition of BMAT

The cellular composition of BMAT explants contributes to meaningful interpretations of tissue activity but this information is often not reported. Without this information, it is not possible to identify the cellular source of secreted factors (e.g., cytokines, adipokines, chemokines) that may be expressed by BMAds, stromal or hematopoietic cells (Zhang et al., 2019; Templeton et al., 2015). This concern is also valid for extramedullary WAT where adipocytes occupy up to 90 % of the tissue volume but often <25 % of the overall cell population (van de Peppel et al., 2017; Goyal et al., 2018; Lee et al., 2013). The cellular heterogeneity of WAT has been defined at the molecular level by snRNA-seq analysis (Emont et al., 2022; Sarvari et al., 2021). Even if BMAT morphologically appears to be predominantly occupied by adipocytes, the medullary stromal and hematopoietic cells can be more abundant. Data on the purity of isolated BMAds and absence of contaminating non-adipocytic cells are instrumental for molecular and functional studies in general, and specifically for the analysis of pro-inflammatory cytokines and stromal factors produced by BMAds (Fan et al., 2017; Liu et al., 2011a; Miggitsch et al., 2019; Ferland-McCollough et al., 2018). Absence of data on the purity of the isolated cell population limits interpretations on the intrinsic ability of BMAds to produce secreted factors (Tencerova et al., 2018; Zhang et al., 2019; Wang et al., 2023a).

Assessing the presence of mature BMAds in BMAT isolates by only measuring adipokine gene expression could introduce bias, because the mRNA levels of typical adipocyte-related genes are low and quite variable in BMAds (Table 2). BMAT adiponectin secretion may also vary depending on the skeletal site (Scheller et al., 2016a). Moreover, several recent studies revealed that not only mature BMAds but also mesenchymal stromal cells, skeletal stem/progenitor cells and adipocyte progenitors residing in BM express ADIPOQ (Zhang et al., 2021; Inoue et al., 2023; Dudakovic et al., 2014; van de Peppel et al., 2017; Zhong et al., 2020; Baccin et al., 2020; Palmisano et al., 2022; Palmisano et al., 2024). In this context, future studies would improve by standardization of isolation procedures and stringent validation of robust biomarkers (e.g., mRNAs or proteins) that define specific cell types in BMAT.

Because there are differences in the cellular composition of rBMAT and cBMAT, the outcome of omics analysis of unfractionated BMAT tissue and/or mixed cell populations derived from BMAT is influenced by the relative presence of rBMAT and cBMAT in marrow specimens. This concern applies not only to transcriptomic analyses (e.g., *LEP*; see Table 2 and (Craft et al., 2019)) but also to proteomics and lipidomics. For example, early rabbit studies compared the FA composition between the entire BM extract and isolated BMAds derived from distinct skeletal sites that differ in the presence of BMAds. While the global FA composition was similar, the FA saturation index for isolated adipocytes and BMAT from BMAd-poor sites was underestimated. The latter finding suggests that the presence of other cell types interferes with the FA measurement (Tavassoli et al., 1977). Moreover, it is well accepted that triglycerides (TGs) are mostly stored in BMAds, but other specific lipid species such as phospholipids, sphingolipids, ceramides, eicosanoids and cholesterol derivatives can also be found in hematopoietic stem cells or the many hematopoietic BM cells (Raza et al., 2021; Tall and Yvan-Charvet, 2015). The presence of lipids from non-adipocytic cell types could confound the exact assessment of contributions by BMAds and bias interpretations.

#### 3.3.3 Assessment of viable cells in BMAT/BMAd specimens

Determination of the viability of BMAds would be beneficial for a better interpretation of experimental data. For example, omics analyses and functional tests applied to BMAds often use extramedullary white adipocytes as a standard reference. Yet, direct comparison may be difficult because the isolation method (and in particular collagenase treatment) may affect cell viability of BMAds (Lucas et al., 2021; Robino et al., 2020). Variability in cell viability can also be observed between different preparations of BMAds within the same study, in particular when using clinical samples (Fazeli et al., 2021; Miggitsch et al., 2019). Therefore, assessments of the viability of isolated BMAds should be implemented when feasible. Useful tests include evaluation of classic Trypan Blue exclusion by light microscopy (Hathaway et al., 1964), nuclear counts and morphology upon DAPI staining (Lin et al., 1977), Annexin V staining for apoptotic cells (Koopman et al., 1994), live/dead staining by fluorescence microscopy (Bayyari et al., 1990), and propidium-iodide staining with assessment of diploid versus non-diploid DNA content by flow cytometry (Crissman et al., 1979).

Among the available techniques, assessment of cell viability by Trypan Blue staining is easily performed on most cell types, except for adipocytes because these cells are large, more fragile and have a relatively small cytoplasmic volume. Flow cytometry can be challenging with fragile adipocytes, although suitable flow cytometry methods have been reported for these cells (Majka et al., 2014). Results from one or more of these assays will help in assessing the fraction of viable versus non-viable cells in BMAd preparations.

#### 3.3.4 General recommendations for experimentation with BMAT/BMAds

To ensure proper interpretation, the cellular composition of BMAT and isolated BMAds should be carefully evaluated in relation to the research question. It is essential to first consider and report the skeletal site of sampling, including the anatomical location within the skeleton (appendicular or axial), the specific skeletal segment (e.g., femur), and the anatomical area within the skeletal segment itself, especially for long bones (diaphyses, proximal or distal metaphysis or epiphysis). Moreover, rBMAds and cBMAds are variably distributed in different skeletal segments depending on the presence of hematopoietic cell types. Thus, it is informative to characterize BMAd prevalence among the other BM cells by histology. In addition, evaluation of viability or functionality should be considered for quality control and simultaneous validation that the cell population contains an adequate fraction of viable cells. The number of recoverable BMAds is often low and does not always allow for validation of each individual sample. If validation of the same sample is not possible due to low yields, then the procedure must be clearly described and the authors should acknowledge that there may be impurities in the BMAd preparations that could confound the results. Quantifiable parameters to describe the homogeneity of BMAd samples require further development and prioritization within the community of BMAT investigators.

## 4 Ex vivo culture of BMAT explants and BMAds

The increasing interest in understanding the functional role of BMAds in health and disease is paralleled by the expansion of available techniques for isolation and characterization of BMAT and BMAd samples. Yet there are few published ex vivo studies that employ BMAT explant or BMAd cell cultures. The scarcity of studies is at least partially due to the absence of standardized approaches that address a number of well-known difficulties associated with primary adipocyte cultures, such as adipocyte buoyancy, delipidation, phenotype loss and hypoxia, which prevent the maintenance of their functional potential during the time-course in culture.

### 4.1 Ex vivo monocultures of BMAT explants and primary BMAds

Short-term BMAT explant cultures (<24 h) are useful for examining the secretory phenotype or metabolic function of BMAT and BMAds. For example, cultures of BMAT and WAT explants from rabbits and humans have been cultured for up to 6 h (1–4 h for rabbit explants and 0.75–6 h for human explants) (Cawthorn et al., 2014). This study analyzed conditioned media to assess adiponectin secretion by BMAT in relation to increased levels of serum adiponectin upon calorie restriction in vivo. BMAT plugs from mouse distal tibia have been cultured for up to 4 h to analyze glycerol and non-esterified FA concentrations in conditioned media as a measure of lipolysis upon induction by forskolin (Li et al., 2022a). Primary human BMAds have been subjected to a short-term, 2-h culture in the presence or absence of isoprenaline for a direct comparison of the lipolytic activity of BMAds and paired subcutaneous adipocytes (SCAds) (Attane et al., 2020). Extensive explant and cell-based studies used 3-day cultures of BMAds for a functional comparison of adipocytes from human femoral BMAT and adipocytes in subcutaneous WAT of the thigh (Miggitsch et al., 2019). These two types of adipocyte cultures were incubated for three days in the presence or absence of adipocyte differentiation cocktail. Even though FACS analysis can affect the cellular integrity of fragile adipocytes (Miggitsch et al., 2019; Majka et al., 2014; Hagberg et al., 2018), cell suspensions were analyzed by FACS, transcriptomic and ELISA approaches, providing insights into lipid uptake and formation, secretion of adipokines and cytokines, as well as pro-inflammatory and ROS-generating processes (Miggitsch et al., 2019). Another representative recent study examined subchondral BMAT lipolysis and BMAd morphology in clinical specimens of carpometacarpal and distal interphalangeal joints in osteoarthritis patients (Maniglio et al., 2025).

### 4.2 Ex vivo co-cultures of BMAds and hematopoietic cells

BMAds interact with multiple cell types within the BM, and this multi-directional crosstalk has the potential to affect their phenotype and function, as well as those of their neighboring cells. Ex vivo explant approaches that can mimic the in vivo microenvironment allow for examination of cell-cell interactions at both functional and molecular levels. One strategy is the use of explant-derived conditioned media, as recently reported in rhesus macaques (Robino et al., 2020). Here, the contribution of BMAT to hematopoiesis was examined using ex vivo BMAT tissue collected from the femur of adult male rhesus macaques. The BMAd-enriched fraction was cultured for 48 h to obtain conditioned media, which was then utilized for characterization of the cellular secretome and for testing of BMAT effects on proliferation and differentiation of hematopoietic stem/progenitor cells (Robino et al., 2020). An example of a sophisticated approach to study the contribution of BMAds to hematopoietic stem cell (HSC) survival was the adaptation of a long-term culture initiating cell (LTC-IC) system to establish a 5-week co-culture model using primary BMAds isolated from the femoral head of hip surgery patients (Mattiucci et al., 2018). Specifically, BMAd suspensions were established as ceiling cultures (i.e., feeder layer composed of a single cell type or mixed culture with BMSCs) and grown in the LTC-IC with CD34^+^ HSCs from healthy BM. A role for BMAds in supporting HSC maintenance, proliferation and differentiation was demonstrated and confirmed by gene expression profiles of hematopoiesis-associated pathways. These studies establish the feasibility of utilizing ex vivo explant cultures to study cell-cell BMAd interactions with neighboring cells. One important consequence of attachment to plastic in ceiling cultures is that adipocytes lose their round morphology and may undergo architectural modifications that affect their phenotype and function. Unbiased analyses that compare ceiling BMAds with mature BMAds using transcriptomic, proteomic, or lipidomic studies will be necessary as a foundation for adopting the ceiling culture model as a new standard culture method and comparing it to other culture approaches.

### 4.3 Ex vivo culture of BMAd-containing bone explants with tumor cells

Bone provides a microenvironment for a number of hematological malignancies and is the site of metastasis for several solid tumors (e.g., breast, prostate). Accruing evidence indicates that progression, survival and therapy evasion of cancers that grow in bone can be significantly impacted by BMAT (Otley and Sinal, 2022). Therefore, there is an emerging need for developing approaches that accurately model the in vivo BMAd-tumor cell interactions in an ex vivo setting. The ability of breast cancer cells to colonize BMAT compartments has been studied using human cancellous bone explant fragments (isolated from the proximal region of femoral heads following hip replacement surgeries) (Templeton et al., 2015). Bone tissue fragments in this 48-h co-culture system were shown to maintain their intact mineralized structure and marrow compartments. This technique provided data that successfully demonstrate the preferential, directed migration of tumor cells towards BMAds in bone. It is evident that the bone explant model represents a very intricate environment, where other cell types, in addition to BMAds, may secrete factors that favor colonization of tumor cells. This colonization may confound evaluation of direct BMAd contributions to tumor cell pathology. More importantly, bone explants represent a very physiologically relevant model that recapitulates the complexity of the BM microenvironment and allows examination of interactions between BMAT and other cell types in bone, including metastatic tumor cells.

### 4.4 Alternative methods for ex vivo culture of BMAds and tumor cells

A number of other models, including long-term, controllable 3D culture approaches have been developed to date as the means to study BMAd interactions with tumor cells and other cell types (Fairfield et al., 2019). These models have one obvious limitation in that they employ BMSC-differentiated adipocytes as opposed to primary adipocytes (Hernandez et al., 2022). However, existing protocols that utilize primary cultures of adipocytes from sites other than BM could potentially be adapted for BMAT/BMAd explant cultures. Detailed methods for preparing and culturing tissue explants or isolated adipocytes from various WAT depots are available (Carswell et al., 2012). Other protocols worth highlighting involve membrane-cultured adipocytes aggregate cultures (MAAC) that are composed of freshly isolated mature adipocytes underneath permeable membrane inserts with small-pores (Harms et al., 2019). Adipocytes cultured in this manner over the course of two weeks maintain gene expression and functional profiles close to the starting adipocytes. These cultures are suitable for gain- or loss-of-function analyses and drug screening studies and could provide an excellent model for studying BMAd-microenvironment interactions. A novel 3D model in which mammary adipocytes are embedded in a fibrin matrix has been developed to study the metabolic crosstalk between mammary adipocytes and breast cancer cells (Rebeaud et al., 2023). This model homogenously distributes adipocytes and prevents their delipidation, while maintaining adipocyte integrity, size and lipolytic activity for up to five days. Co-cultures with breast cancer cells are possible without altering the matrix and permits monitoring of FA transfer between cells and lipidomic changes that influences breast cancer aggressiveness in an obesity-dependent manner.

## 5 Molecular analyses of isolated BMAds and precursors

Recent molecular studies have highlighted distinct biochemical and physiological properties of BMAds isolated from various bone parts. These skeletal site-specific differences in lipid composition, gene expression and secretory profiles of isolated BMAds require further consideration, and can be examined both in vivo (Scheller et al., 2015) or ex vivo (Ehnert et al., 2023). Other insights have been obtained from studies on lipid composition in BMAds from WAT (Liu et al., 2011a; Miggitsch et al., 2019) and BAT (Suchacki et al., 2020) in mouse (Liu et al., 2011a), rat (Scheller et al., 2015) and human specimens (Poloni et al., 2013; Miggitsch et al., 2019). Proteins secreted by BMAds also provide important insights into the endocrine or paracrine functions of BMAds (Sulston and Cawthorn, 2016; Scheller et al., 2016b).

Molecular analyses will help with defining the differences between regulated or constitutive BMAT. While this distinction is useful for conceptualization of current findings, we appreciate that it may represent a binary over-simplification of a gradual continuum of phenotypic states. For example, certain transgenic modifications impact cBMAT in the tibia but not in caudal vertebrae of mice (Lovdel et al., 2024; McIlroy et al., 2018), indicating further heterogeneity among cBMAd subtypes. Moreover, recent human genomic studies using data deposited in the UK Biobank revealed that BMA-associated genetic variants differ not only between BMA in the spine and femur, but also between different femoral regions (Xu et al., 2025). Thus, a binary categorization of BMAT subtypes is almost certainly an over simplification. Rather, a broad range of BMAT subtypes may exist with rBMAds and cBMAds at different ends of the spectrum, and these subtypes may exhibit considerable plasticity and perhaps have the ability to transform into other subtypes.

Given the heterogeneous nature of BMAT, comparative studies between rBMAds and cBMAds must report on specific locations from which these cells are isolated. To date, there are no unique cellular markers that discriminate between these two cell types. Therefore, the identification of such markers is essential for exploring potential functional differences between these BMAT subtypes. Advanced techniques like single-cell sequencing and spatial transcriptomics may prove useful in this endeavor. Single-cell level omics approaches can provide an in-depth understanding of the heterogeneity of BMAds and delineate the spectrum of cellular morphologies and biochemical pathways that dictate BMA pathophysiology in health and disease.

State-of-the-art molecular analyses by transcriptomic, proteomic or lipidomic profiling are available to characterize BMAds and BMAT from both human and rodents. These studies are typically complemented with loss-of-function and gain-of-function experiments using cultured BMAds or co-culture experiments with other cell types. Bulk RNA-seq and microarrays are commonly used and effective to capture gene expression profiles in many tissues and cell populations including BMAds and BMAT from species such as humans, rabbits, and mice (Suchacki et al., 2020; Liu et al., 2011a; Aaron et al., 2021; Craft et al., 2019). The scarcity of BMAds in mice and other small mammals complicates BMAd isolation and purification of BMAds from such species. However, given the abundance of BMAds within the distal tibia, utilizing distal tibia BM plugs as an alternative to isolated BMAds for bulk RNA-seq may offer valuable insights into BMAd gene expression profiles in small mammals (Li et al., 2022a).

In the sections below, we discuss specific omics studies using BMAT or BMAd samples. General biochemical recommendations for molecular analyses of BMAT or BMAd samples are that specimens should be isolated rapidly and brought to a low temperature (<4 °C) as soon as possible. Chemical inhibitors that can block degradation of biomolecules by potent stable enzymes (e.g., ribonucleases/RNases, proteases, phosphatases) are useful, as long as they do not interfere with subsequent analyses. However, the most effective method for maintaining molecular integrity is to decrease temperature and increase speed that, respectively, reduce the speed of degradation and the time that degradation is allowed to proceed. Slower degradation plus less time to degrade results in a molecular profile more similar to that occurring in vivo. For this reason, tissue and cell harvests from in vivo models should ideally be done in teams such that manual handling is not rate-limiting for efficient sample processing. Following isolation, calibrated volumes or equal cell numbers of adipocytes can be frozen in liquid nitrogen and samples kept at −80 °C until use. Depending on the type of future analyses, samples can be stored in stabilizing media to improve stability for long-term storage (Lucas et al., 2021). Shipment of RNA samples to collaborators for further analysis is best done as cell lysates in TRIzol™ on dry ice (or a comparable ‘double-kill’ method to block RNases).

### 5.1 Transcriptomic analyses

Differences in gene expression between rBMAds and cBMAds remain poorly understood, but some insights have been gained from extensive comparisons of gene expression between primary purified BMAds and adipocytes from WAT (Liu et al., 2011a; Miggitsch et al., 2019) and BAT (Suchacki et al., 2020) in mouse (Liu et al., 2011a), rat (Scheller et al., 2015) and human specimens (Poloni et al., 2013; Miggitsch et al., 2019). One small-scale study directly compared expression of major adipogenic transcriptional factors in purified rBMAds and cBMAds from rats. These studies indicated that C/EBPα (*Cebpa*) and C/EBPβ (*Cepbb*) are selectively elevated in cBMAds compared to rBMAds (Scheller et al., 2015). This result suggests that constitutive versus regulated adipocyte populations employ alternative mechanisms for transcriptional regulation.

For gene expression analyses, different types of adipocytes can be used including cBMAds or rBMAds; purified cells versus BMAd-enriched fractions that remain associated with other cells; or cells embedded in either rBMAT or cBMAT. Standard isolation procedures are utilized to obtain BMAd-enriched fractions by centrifugation or BMAds isolated by collagenase digestion. Because cBMAds and rBMAds display differences in FA content, this phenotypic difference in lipidome expression may be reflected by expression of adipose-related enzymes. Similarly, hematopoietic and immune cells possess considerable amounts of phospholipids and eicosanoids that may affect mRNA expression signatures. In addition, other tissue resident cells (e.g., endothelial cells, smooth muscle cells, nerve cells) provide extraneous gene expression profiles that can significantly modify the interpretation of both transcriptome gene expression profiles and the lipidome.

Transcriptomics studies have been performed with mouse BMAd-enriched fractions that were obtained by flushing BM from both femur and tibia, and separating the floating BMAds by centrifugation (Liu et al., 2011a). Collagenase-digested human BMAds are isolated from the femoral head or proximal femoral diaphysis or from trabecular bone of the proximal femoral metaphysis of patients undergoing hip-replacement surgery (Suchacki et al., 2020; Mattiucci et al., 2018; Miggitsch et al., 2019). Both cBMAds and rBMAds can be harvested from several skeletal sites in rats to assess site-specific differences (Scheller et al., 2015). The purity of adipocyte samples can be increased by removal of residual contaminant cells and affinity purification using cell type-selective antibodies coupled to magnetic beads (Miggitsch et al., 2019). BMAds are rapidly frozen and processed for RNA extraction, to support analysis by RNA-seq, microarrays and/or RT-qPCR.

RNA-seq data have been acquired for human epiphyseal and metaphyseal MSCs and BMAds that were isolated from OA patients. The MSCs were differentiated into osteoblasts and adipocytes, and then compared to BMAds and SCAT adipocytes (Zapata-Linares et al., 2025). Gene ontology analyses of these transcriptomes suggest that metaphyseal OA-BMAds may have adapted to support hematopoietic stem cell differentiation, while epiphyseal OA-BMAds appear to have more osteogenic potential and express biomarkers linked to bone mineralization and remodeling. The combination of GWAS meta-analyses, transcriptome-wide association studies, and deep learning approaches of clinical images (MRIs) of large public databases (e.g., UK Biobank) is effective for gaining translational insights and understand molecular, biological and pathophysiological parameters that modulate BMAT activity (Xu et al., 2025).

### 5.2 Single-cell and spatial sequencing approaches

#### 5.2.1 Single-cell RNA-sequencing (scRNA-seq)

In purified BMAds or BMAT samples, the presence of hematopoietic cells, vascular cells, and mesenchymal cells can skew results and interpretation of bulk RNA-seq analyses. Single-cell RNA sequencing (scRNA-seq) can overcome this limitation, but requires fluorescence-activated cell sorting (FACS) to separate individual cells within a suspension. Single adipocytes are not easily isolated by FACS due to their relative size, fragility and the presence of lipids. Therefore, FACS analysis has not been widely used for isolation or evaluation of BMAds (Miggitsch et al., 2019). Nevertheless, key adaptations of FACS protocols that have been successfully implemented to improve cell yields of BMAds from WAT are the use of a large nozzle, application of low sheath pressure and enhancement of the detection of larger events (Hagberg et al., 2018).

#### 5.2.2 Single-nucleus RNA sequencing (snRNA-seq)

Given the fragility of adipocytes, snRNA-seq is an effective novel method to capture gene expression in nuclei isolated from BMAT and/or BMAds. The combination of sc- and snRNA-seq was applied to investigate the transcriptome of SCAT versus visceral adipose tissue (VAT) in human and mouse samples (Emont et al., 2022). Systematic omics studies like this are particularly useful and provide critical benchmarks for the field. Thus, the application of snRNA-seq to BMAT and BMAds holds great promise to reveal new fundamental and translational insights.

#### 5.2.3 Spatial transcriptomics

To overcome the intrinsic fragility of isolated adipocytes and to understand the heterogeneity of BMAT between species across different skeletal sites, spatial transcriptomics can be applied as a versatile tool to understand gene expression in specific regions of interest within a tissue. Spatial transcriptomics involves capture and detection of transcripts in dots within a matrix across histological sections. This molecular approach is analogous to a pointillism painting, allowing the establishment of a genetic map or cluster with cell population and tissue specificity (Langin, 2021). Spatial transcriptomics RNA-seq can be performed on any histological section of adipose tissue. Subcutaneous abdominal WAT from ten individuals has been analyzed using the 10× Genomics Visium Spatial Gene Expression platform (Backdahl et al., 2021). This study revealed the presence of three distinct adipocyte populations (i.e., AdipoLEP, AdipoPLIN, and AdipoSAA) that were selectively enriched in genes relating to different metabolic processes: respectively, extracellular matrix (ECM) and cell-cell interactions (AdipoLEP), leptin secretion and iron metabolism (AdipoPLIN), and retinol metabolism (AdipoSAA) (Backdahl et al., 2021). The relevance of this data set was validated by comparison with snRNA-seq and scRNA-seq data from VAT and SCAT (Massier et al., 2023). Spatial transcriptomics have also been performed on mouse BAT to define the spatial patterning of adipocyte subpopulations in mice subjected to primary or secondary cold exposure at 4 °C (Lundgren et al., 2023). We anticipate that applying these techniques to BMAT across various species will reveal specific patterns of BMAd in relation to different skeletal locations and pathophysiological contexts.

### 5.3 Epigenomics

As a highly dynamic tissue, adipose tissue exhibits gene expression changes in response to different conditions including diet, exercise, cold and disease. These modulations are, in turn, regulated by epigenetic modifications: reversible modifications to chromatin that define, at least in part, the chromatin structure and the level of gene expression without altering the DNA sequence. Well-studied epigenetic modifications include DNA methylation, histone post-translational modifications, incorporation of histone variants, noncoding RNA regulation of target genes, and the role of chromatin remodeling enzymes that modify the interactions between DNA and histone complexes. Extensive studies have been carried out to delineate the dynamic epigenetic regulation of adipogenic differentiation of white adipocytes (Mikkelsen et al., 2010; Nanduri et al., 2022) and brown adipocytes (Brunmeir et al., 2016), as well as adipogenic lineage commitment of BMSCs (Wang et al., 2023b). Because there is a paucity of studies on the epigenomic mechanisms by which mature BMAds respond to local or systemic pathophysiological cues, future studies should address this major frontier in BMAT research.

### 5.4 Proteomics

Relying solely on gene expression techniques such as RNAseq or spatial transcriptomics in adipose tissue research provides an incomplete picture, because increased transcript levels do not necessarily result in increased translation of the protein (e.g., miRNA suppression or protein degradation), nor do transcriptomics account for functional properties of proteins influenced by post-translational modifications. Proteomics analysis offers a valuable complement by directly validating protein levels (by spectral counting) and assessing protein modifications. Proteomics supports protein discovery in cell lysates independent of prior knowledge of pathways and in principle can detect any protein within the cells using quantitative LC-MS and bioinformatics (Graves and Haystead, 2002). Proteomics combined with high-throughput immunofluorescence microscopy methods can create comprehensive 3D maps of cells and tissue, indicating where proteins are located and what interactions occur between them.

Several studies have utilized proteomics to study proteins present in WAT, VAT and SCAT biopsies across different pathologies (type II diabetes, hypertension and obesity). One of these studies showed that the adipokine Omentin-1 (Intelectin-1, ITLN1) correlates with diabetes in VAT, while levels of proteins from the endoplasmic reticulum and stress-related proteins are elevated in SCAT (Hruska et al., 2023). Proteomics has also been applied on WAT biopsies derived from studies on the effects of high-intensity interval training (HIIT) in a cohort of 48 patients including lean individuals, obese individuals and those with type II diabetes (Larsen et al., 2023). The proteome of BAT has been studied in mice subjected to a high-fat diet (HFD) for 22 weeks (Li et al., 2015). Compared to WAT, to date only two studies performed proteomic analyses with BMAds (Robino et al., 2020; Attane et al., 2020). The proteomes of adipocytes isolated from human femoral BM, obtained from patients undergoing hip surgery, were compared to subcutaneous adipocytes from the same patients (Attane et al., 2020). Translationally relevant studies on primates used rhesus macaques for analysis of secreted proteomes in the femur of BMAT using LC-MS/MS (Robino et al., 2020). Collectively, these studies reveal the utility of proteomics in studies to understand the molecular changes in BMAT in relation to metabolic disorders and exercise.

### 5.5 Lipidomics

Lipids play diverse functional roles within BMAT and the use of lipidomics can provide new insights into their composition and function. Lipidomics is a type of metabolomics focused on the large-scale analysis of lipids, specifically aiming to determine cellular lipid species. Due to high chemical diversity and complexity, the entire lipid spectrum is not yet fully known. Consequently, there are only a limited number of studies that analyze the lipid composition analysis of BMAds.

Beyond the biochemical studies presented below that examine lipids ex vivo in isolated BMAT, in vivo proton magnetic resonance spectroscopy (H-MRS) analyses in mammals (e.g., human, rat) permit evaluation of overall lipid content in living subjects. One H-MRS study showed that rat rBMAs has a higher proportion of saturated FAs, while cBMAs have increased unsaturated lipid species (Fig. 2D) (Scheller et al., 2015). The same study showed that lipid saturation is also higher in human rBMA-enriched skeletal regions (e.g., proximal femoral metaphysis, mid-femoral diaphysis, tibial diaphysis) compared to cBMA-enriched sites (e.g., distal tibia) (Scheller et al., 2015). Thus, both biochemical and biomedical imaging analyses ex vivo and in vivo provide complementary insights into the lipid composition of BMAT.

#### 5.5.1 Untargeted lipidomics

The lipid profile of isolated BMAds can be characterized using quantitative LC-MS, which is the primary technique to study lipid composition. LC-MS includes various modes based on different separation techniques, with one major method being Reversed Phase (RP) separation (RPLC-MS), which is used for non-polar and mid-polar molecules. The chromatographic separation provides analytical information for subsequent MS and tandem MS (MS/MS) (Harrieder et al., 2022; Dai et al., 2023). Different protocols can be used to purify lipids, including extractions with cold-methanol, methanol:dichloromethane, methyl tert-butyl ether (MTBE), and 10 % methanol in the absence of internal standards (Gonsalves et al., 2020; Benova et al., 2023).

Four different LC-MS platforms have been used for lipid profiling in BM, bone powder and plasma samples (Benova et al., 2023), including (a) lipidomics of complex lipids using (RPLC-MS) in positive ion mode, (b) lipidomics of complex lipids in RPLC-MS in negative ion mode, (c) metabolomics of polar metabolites using hydrophilic interaction chromatography with mass spectrometry (HILIC-MS) in positive ion mode, and (d) metabolomics of polar metabolites using RPLC-MS in negative ion mode (Benova et al., 2023). These approaches resulted in the discovery of >900 metabolites in different mouse tissues (Benova et al., 2023). In a separate study, tandem liquid chromatography-mass spectrometry (LC-MS/MS) was used to analyze total lipid content extracted from human isolated BMAds and subcutaneous adipocytes, identifying 818 lipid species from 15 different lipid classes (Attane et al., 2020). Another study used BM plasma from patients with monoclonal gammopathy of undetermined significance (MGUS) and multiple myeloma (MM), identifying >1000 metabolites by an untargeted ultra-performance mass spectrometry (UPLC-MS/MS) (Gonsalves et al., 2020). These studies collectively indicate that lipidomic profiling offers novel approaches to characterize lipid species and their pathophysiological functions in BMAT and/or BMAds.

Published methods are available for untargeted lipidomic profiling of isolated BMAds (Attane et al., 2020) that are similar to previously reported protocols for adipocytes and adipose tissue (Lange et al., 2021). Human BMAds can be isolated from yellow BMAT (e.g., harvested from patients undergoing hip surgery) using collagenase digestion and rapidly frozen at −80 °C for future analysis. Total lipids are extractable with methyl-tert-butyl ether (MTBE) which permits isolation of both lipids and proteins from the same sample (Attane et al., 2020), or using other procedures such as the Folch or Bligh-Dyer methods described for WAT (Lange et al., 2021). Ehnert and colleagues compared total FA composition based on fatty acid methyl ester gas chromatography–mass spectrometry (FAMEGC-MS) in samples from plasma, red and yellow BM obtained from femoral heads (Ehnert et al., 2023). Their data demonstrate that specific FA compositions in red and yellow BM correlate statistically with bone mineral density (BMD), suggesting that FA composition is a biomarker and potential modulator of bone homeostasis.

At a technical level, for a typical adipocyte suspension (total volume 600 μl), 100 μl of adipocyte suspension is sufficient for efficient global lipid analyses (Attane et al., 2020) if only lipidomic analysis is planned. The remainder of the adipocyte suspension (500 μl) can be used for proteomic studies. The majority of lipids detected in both white adipocytes and BMAds by tandem liquid chromatography-mass spectrometry (LC-MS/MS) are triglycerides (TGs), which account >90 % of the total lipid content; the other major sample components are diglycerides and phospholipids (Attane et al., 2020; Lange et al., 2021). Total TG content was also determined in rabbit femoral BM plugs (Cawthorn et al., 2016). In these studies, samples were frozen on dry ice before cryopulverization in liquid nitrogen and total lipid was then extracted using the Folch method followed by TG quantification using a commercial assay kit (Cawthorn et al., 2016).

#### 5.5.2 Targeted lipidomics

FA composition can also be assessed by targeted lipidomics as was done for human BM aspirates from pediatric vertebrae (BMAd-enriched fraction) (Whitney et al., 2021; Whitney et al., 2020), human femoral and tibial BMAT (Griffith et al., 2009b), human iliac crest and femoral head BMAd-enriched fraction (Tratwal et al., 2022), human red and yellow BM (Ehnert et al., 2023), femoral and tibial BM in mice (Bartelt et al., 2017), as well as rat cBMAds and rBMAds (Scheller et al., 2015). For this analysis, tissues are rapidly harvested and frozen, or digested to generate BMAds, which are then frozen until analysis. Lipids are then extracted with Bligh-Dyer methods and FAs converted into the corresponding FA methyl esters (FAME). These compounds are then further purified using thin-layer chromatography and analyzed by gas chromatography with a flame-ionization detector. This type of lipidomics analysis revealed that cBMAds and rBMAds have distinct lipid profiles and that cBMAds have higher levels of unsaturated FAs. Importantly, less-abundant lipids such as phospholipids, cholesterol derivatives or eicosanoids are difficult to detect due to the abundance of TGs (Scheller et al., 2015). Hence, detection of rare lipids may necessitate protocol optimization to specifically extract non-TG lipids using thin layer chromatography or solid phase extraction.

Focused lipidomic data (e.g., free fatty acids and glycerol) were obtained from BMAT from patients with hand osteoarthritis (OA). The results indicate that BMAT lipolysis regulates osteoblast activity in hand OA, and that the lipolytic activity of BMAT depends on the condition of the surrounding tissues and the anatomical location within the hand (Maniglio et al., 2025).

### 5.6 Functional assays

To assess adipocyte functions, only fresh adipocytes can be used as freezing compromises cell membranes and results in lysis of adipocytes. Indeed, relatively few studies have investigated BMAd function because the fragility of these cells causes rupture during flow sorting or digestion, such that the yield of isolated adipocytes is often too low (Attane et al., 2021). Precautions to limit adipocyte loss during digestion have been reported (Attane et al., 2021; Lucas et al., 2021), including addition of 0.25 % sodium citrate during the digestion step to reduce cell loss due to clotting (Scheller et al., 2015).

#### 5.6.1 Secreted factors from BMAT explants and BMAds

Because BMAds are secretory cells, they produce not only FAs but also bioactive proteins such as ADIPOQ, LEP, stem cell factor (SCF or KITLG) and RANKL (TNFSF11) (Sulston and Cawthorn, 2016; Scheller et al., 2016b). The secretory profiles of rBMAds and cBMAds and their responsiveness to endocrine and metabolic stimuli represent attractive questions that remain to be addressed. Beyond candidate secreted proteins (‘the usual suspects’, e.g., ADIPOQ, LEP), secretory profiles involving less well-known proteins can be obtained by proteomics (e.g., in conditioned media of BMAds) or by gene ontology analysis of RNA-seq data (e.g., a ‘virtual secretome’ of mRNAs encoding secreted factors).

One functional outcome of BMAT studies is the determination of the presence of secreted factors in conditioned media from BMAT explants, isolated BMAds or BM plugs to assess the function of adipocytes. For example, increased adiponectin secretion in BMAT versus WAT represents a physiological difference that has been reported in rabbit and human samples (Cawthorn et al., 2014; Miggitsch et al., 2019). Different biological source materials and ex vivo culture methods have been applied to assess proteins secreted from BMAT or BMAds in relation to other tissues and cell types. Culturing explants and cells in media that is serum free and/or synthetic is preferred over culture medium that contains serum, to avoid zoonotic contributions or interference from serum-intrinsic factors.

In one set of experiments, rabbit BMAT and WAT were dissected into explants and incubated in Krebs Ringer Bicarbonate buffer to permit protein secretion, followed by analysis of adiponectin levels (normalized to total protein) by immunoblotting and silver staining of conditioned media (Cawthorn et al., 2014). A second set of experiments involved human explants of BMAT and WAT isolated from patients undergoing orthopedic surgery (e.g., knee amputation), followed by culture in serum-free Dulbecco's modified Eagles' medium (Cawthorn et al., 2014). Thirdly, human adipocytes isolated from the femoral head and WAT were incubated in RPMI medium supplemented with 10 % serum. Conditioned media was then collected and frozen for subsequent analysis of adipokine secretion (e.g., ADIPOQ, LEP, TNFα/TNF, IL6, and IL8) using commercial ELISA kits (Miggitsch et al., 2019).

#### 5.6.2 Metabolic activity in BMAT explants and BMAds

Metabolic function can also be studied in BMAT explants or isolated BMAds incubated for short times and treated with or without isoprenaline or forskolin to evaluate stimulated and basal lipolysis by measuring glycerol and FA release (Scheller et al., 2019; Zakaria and Shafrir, 1967; Li et al., 2022a; Attane et al., 2020; Tran et al., 1981). In addition, BMAT explants can be treated with isoprenaline to determine ex vivo cAMP content. For longer incubation times (more than 24 h), BMAds have to be cultured in specific conditions to preserve their physical and chemical features of the adipocytes. Indeed, as for white adipocytes (Rebeaud et al., 2023), freshly isolated adipocytes cannot be kept longer than 24 h in suspension, presumably because of anoikis (cell death through lack of adherence to a substrate). To avoid loss of adipocytes, these cells should be maintained in 3D culture. Alternatively, BMAT explants or bone fragments containing adipocytes can be used as described for co-culture experiments with cancer cells (Templeton et al., 2015).

The key point for experiments aimed at studying functions of BMAds is that proper experimentation requires optimized cell isolation protocols to obtain purified adipocytes and to maintain cell integrity while avoiding lysis during incubations. Because collagenase digestion can impact viability and functionality of cells (Lucas et al., 2021), (Robino et al., 2020) it is possible that collagenase-digested BMAds and centrifuged BMAd-enriched fractions differ in biological properties. The latter remains to be examined in side-by-side comparisons.

## 6 Recommendations and guidelines

Isolating high-quality, viable BMAT from animals and humans is the most-critical step for successfully performing a broad range of molecular and functional assays. As summarized in Table 1, BMAT is a heterogeneous tissue based on its source, location and method of isolation, each of which influences its cellular composition and the broad type of BMAT (cBMAT vs rBMAT). These factors can substantially impact the outcome and interpretation of results, and it is therefore critical to fully describe these variables in methods reporting BMAT and/or BMAd isolation protocols. Such reporting is essential to increase the reproducibility of the data and the possibility of sharing samples for future collaborative studies.

BMAd isolation methods in animal models are either based on collagenase treatment or on a quick centrifugation method. Literature reporting on BMAd isolation protocols in animal models does not provide a uniform approach as data are presented using different species (e.g. mouse, rat, rabbit, rhesus macaque) (Table 1), which may reflect different stages of development and regulation of BMAT (Tratwal et al., 2020; Robino et al., 2020; Zakaria and Shafrir, 1967; Craft et al., 2019). As discussed above, BMAd isolation from mice is usually devoid of collagenase digestion (Fan et al., 2017), while BMAd isolation from rat or rabbits (Craft et al., 2019; Tavassoli, 1978) may include collagenase digestion. This variation can complicate interpretation of the data beyond factors such as age, species or sex differences in the study design.

Similar heterogeneity in BMAT isolation protocols is observed in clinical studies (Table 1) and depends on the type of collection method (BM aspirate vs surgery), the skeletal site, the scientific questions being addressed, and the downstream analyses for which the BMAT and/or BMAds will be used. Thus, from the perspective of planning experiments including BMAT and/or BMAd isolation, several critical aspects must be considered (Table 3).

**Table 3:** Experimental considerations and recommendations for studies on BMAT and BMAds.

Parameter	General comments / recommendations	Specific to human samples	Specific to murine samples
Use of collagenase	▪BMAT in mice smaller and with less trabecular network than in humans or bigger mammals, resulting in different isolation methods. ▪Ensure that new batches of collagenase are tested with careful examination of digestion. ▪Be aware that collagenase digestion can affect some BMAd characteristics for metabolic and RNA analysis.	▪Recommended (Table 1): Collagenase digestion reduces potential deficiencies in BMAT amounts and reduces the effects of variability in BMAd content. It is usually performed for comparison with non-medullary adipose tissues. ▪Be aware that collagenase digestion of BMAT is faster than that of SCAT, probably because of the decreased extracellular matrix.	▪Not required: the BM can be conveniently harvested from femurs and tibiae through a simple centrifugation step for further processing without prior collagenase digestion.
Time and transfer	▪The faster, the better! ▪Be careful with temperature fluctuations which can increase the fragility of BMAds.	▪Ensure that all logistical steps from the operating room to the laboratory have been tested and are well-controlled. This will notably include that the surgical team has been informed about protocols for handling tissues (with no clinical value, the requirement of preparation by pathologists) as well as the duration and storage conditions during the transferring process.	▪The speed of isolation is also determined by the efficiency of bone cleaning (muscle removal); any variance in speed may lead to stochastic variation among samples.
Quantity of material	▪This will depend on the source of BMAT, species, age and general pathophysiological condition. ▪The limited amount of BMAT/BMAds can also prevent assessment of tissue and cell purity after the isolation for each preparation.	▪Macroscopic observation can help identify samples more enriched in yellow BM compared to red BM and adapt the isolation strategy ▪Save a small representative aliquot of your initial sample for immunohistology and thus a better characterization of your BMAd isolation yield.	▪The limited amount of BMAT/BMAds usually requires pooling specimens from different bone sites and several individuals which should be clearly stated.
Standardization and reproducibility	▪It is instrumental to document as much as possible - the biological source: bone and site (proximal/distal; rBMAT/CBMAT); main “donor” characteristics; - the used methodology from sampling, transferring, isolation to the data analysis; - the validation process for purity and viability: markers and experimental type; used sample number.	▪Considerable donor-to-donor variation resulting in major intra-group variability that compromises the statistics of inter-group comparisons. ▪Recording of relevant covariables (e.g. age, sex, BMI, co-morbidities) can help to identify confounding variables to control for statistical analyses.	▪Easier for standardization of maintenance and isolation procedures: the validation process may only be performed on a small number of BMAT/BMAd samples. ▪Crucial to report the type of bone and site, the number of pooled samples and animals.
Purity and viability	▪Quantifiable and empirical criteria for inclusion or exclusion remain to be established (section 3.3). ▪It is crucial to document the potential presence of stromal, hematopoietic and bone cells besides typical adipocyte markers in your final samples before analysis and interpretation. ▪The use of tests based on protein analysis (whenever feasible) may be the most relevant.	▪Advised to follow the degree of cell composition from sampling to final isolation for each donor when quantity is sufficient. ▪Save a small representative aliquot of your final sample for immunohistology or in vitro incubation to monitor purity and viability.	▪Remains challenging since requires the whole final isolated BMAd sample to be subjected to immunohistochemistry or in vitro incubation. If possible, a small initial study should be performed for validation purposes.

## 7 Future perspectives

Research into BMA, BMAT and BMAds has grown substantially in the past 20 years (Bravenboer et al., 2019), reflecting increased awareness of BMAds' importance not only in fundamental biology, but also the relevance of this tissue to human health and disease. This groundswell in BMA research is thereby fueling a self-reinforcing positive feed forward loop (‘virtuous cycle’) that has a beneficial effect: by identifying ever-broader pathophysiological implications, the scope of scientific disciplines relevant to BMA continues to expand, attracting new researchers and methods to the BMA field. This growth and diversity are overwhelmingly positive. Nevertheless, this expansion also creates variability in reporting and methodologies, which can hinder the ability to interpret, reproduce, and build upon existing findings. Thus, continued progress requires improved rigor and transparency in reporting and methodologies for BMA research, a goal that this review has sought to address.

Robust methods for the isolation and analysis of BMAT and BMAds are essential if we are to continue establishing their fundamental and translational functions, as well as the molecular mechanisms that underpin them. In this narrative, we highlighted key factors that may impact the properties of isolated BMAT and BMAds and suggest ways to confirm the quality of these samples. However, a clear challenge is that standard baseline markers remain to be established to define the normal (‘healthy‘) state of BMAT and BMAds, and the variables that impact these tissues. It is clear that properties of BMAT and BMAd differ depending on skeletal site, BM cellular heterogeneity, the method of sample isolation, as well as donor demographics and characteristics (e.g. age, sex, health status, comorbidities), each of which may differ widely between studies. Future research must therefore determine systematically how such variables influence BMAT and BMAds.

While the prospect of accounting for all biological parameters seems daunting, new and emerging technologies are well placed to address this biomedical challenge. For example, snRNA-seq and spatial omics methods have the potential to establish the scope and heterogeneity of BMAd subtypes, single-cell based studies will likely cause a paradigm shift beyond the binary (and perhaps overly simplistic) “constitutive” and “regulated” categorization. This concept that there may be a spectrum of BMAds based on recent genome-wide association studies of BMAT in humans (Xu et al., 2025). These state-of-the-art single-cell and spatial omics methods could reveal a continuum of BMAd phenotypes, highlighting including differences between BMAds, extramedullary adipocytes and other BM cell populations, as well as potential variations based on anatomical location and pathophysiological contexts. Sophisticated single-cell studies could also more clearly define molecular hallmarks (‘signatures’) of BMAds. From a methodological perspective, such phenotypic signatures would be extremely valuable in validating the quality of BMAT and BMAds used in future studies. Perhaps more importantly, such hallmarks would also transform understanding of BMAT and BMAd formation and function.

A remaining challenge is how to identify the full spectrum of clinical conditions that are influenced by changes in BMA. In humans, BMA is typically measured using magnetic resonance imaging or spectroscopy (MRI or MRS), methods that are expensive and time-consuming to analyze. Consequently, almost all cohorts for previous human BMA studies have not exceeded 750 participants (Morris et al., 2024; Shen et al., 2014). This relatively modest statistical sample size has limited our ability to understand the clinical implications of BMA. This limitation may soon be surmounted by advances in deep learning (Xu et al., 2025; Liu et al., 2021). Large-scale analyses of MRI data to measure other adiposity traits revealed new pathological associations (Liu et al., 2021). Similar methods have recently been developed for large-scale analysis of BMA, including GWAS analyses (Morris et al., 2024) Their application promises to hugely extend understanding of the pathophysiological relevance of altered BMA, thereby continuing the virtuous cycle and fuelling further growth in BMA research. In this light, the issues addressed in the present paper are particularly timely. We trust that this paper will help support future high-quality research collaborations in this exciting ever-expanding field.

## CRediT authorship contribution statement

**Michaela Tencerova:** Writing – review & editing, Writing – original draft, Conceptualization. **Biagio Palmisano:** Writing – review & editing, Writing – original draft, Conceptualization. **Stéphanie Lucas:** Writing – review & editing, Writing – original draft, Conceptualization. **Camille Attané:** Writing – review & editing, Writing – original draft, Conceptualization. **Kaisa K. Ivaska:** Writing – review & editing, Writing – original draft, Conceptualization. **Léa Loisay:** Writing – review & editing, Writing – original draft, Conceptualization. **Yoshiko M. Ikushima:** Writing – review & editing, Writing – original draft, Conceptualization. **Drenka Trivanovic:** Writing – review & editing, Writing – original draft, Conceptualization. **Alessandro Corsi:** Writing – review & editing, Writing – original draft, Conceptualization. **Adriana Roque:** Writing – review & editing, Writing – original draft, Conceptualization. **Hongshuai Li:** Writing – review & editing, Writing – original draft, Conceptualization. **Friederike Behler-Janbeck:** Writing – review & editing, Writing – original draft, Conceptualization. **Jeroen Geurts:** Writing – review & editing, Writing – original draft, Conceptualization. **Mara Riminucci:** Writing – review & editing, Writing – original draft, Conceptualization. **Izabela Podgorski:** Writing – review & editing, Writing – original draft, Conceptualization. **William P. Cawthorn:** Writing – review & editing, Writing – original draft, Methodology, Conceptualization. **Bram C.J. van der Eerden:** Writing – review & editing, Supervision, Project administration, Conceptualization. **André J. van Wijnen:** Writing – review & editing, Writing – original draft, Supervision, Project administration, Conceptualization.

## Authorship contributions

All authors contributed to the conceptual development, writing and editing of this manuscript. All authors approved the final version of the paper and agreed to authorship.

## Funding

US based investigators that participated in the study were supported in part by funding from the 10.13039/100000002National Institutes of Health through the following awards: R01-AR076357 and R01-AR083398 (HL), R01-CA251394 (IP) and R01–049069 (AJvW).

## Declaration of competing interest

There are no competing interests to declare for any of the authors.

## Data Availability

No data was used for the research described in the article.
